# Harnessing Molecular Recognition for Small‐Molecule‐Mediated Reversible Photochemical Control Over mRNA Translation

**DOI:** 10.1002/anie.202503078

**Published:** 2025-04-22

**Authors:** Shaifaly Parmar, Logan Tenney, Xiao Liang, John T. Routzahn, Christopher D. Sibley, John S. Schneekloth

**Affiliations:** ^1^ Chemical Biology Laboratory Center for Cancer Research National Cancer Institute Frederick MD 21702‐1201 USA

**Keywords:** Aptamers, Chemical biology, Photochemistry, RNA, Translation

## Abstract

Chemical probes that control the function of complex RNA molecules offer unique opportunities to interrogate biological systems. In this study, we demonstrate that a small molecule ligand selectively recognizes and undergoes traceless, reversible photocrosslinking to PreQ_1_ RNA aptamers. This effect is selective and dependent on both the chemical structure and RNA sequence/structure. A homogeneously modified, caged mRNA construct containing a PreQ_1_ aptamer and an eGFP or wild type p53 coding sequence displayed repressed translation in vitro or in cells until irradiated with 302 nm light, resulting in cleavage of the photocage and restoration of translation. This method demonstrates for the first time that aptamer‐based molecular recognition of a small molecule ligand can be used to precisely and photochemically activate the translation of a complex mRNA in cells.

## Graphical abstract

A small molecule ligand undergoes traceless, reversible photocrosslinking to PreQ_1_ RNA aptamer in a selective manner, dependent on both the RNA sequence/structure and ligand chemical structure. A caged mRNA construct incorporating the PreQ_1_ aptamer and an eGFP or p53 coding sequence remained translationally repressed until exposure to 302 nm light, which cleaved the photocage and restored translation in vitro and in cells, enabling precise photochemical control of both wild type and reporter gene expression.

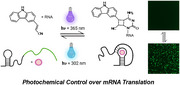

## Introduction

RNA is a structurally complex biopolymer that impacts a multitude of roles in human biology including regulation of gene expression, catalysis of biochemical reactions, metabolism, and immunology.^[^ ^1^, ^2^, ^3^ ^]^ Therefore, chemical methods that enable control over the behavior of RNA molecules to understand their role within biology are in high demand.^[^ ^4^, ^5^ ^]^ To this end, covalent, photoreversible ligand‐aptamer pairs may provide an attractive approach to exercise conditional control over gene expression with spatial and temporal resolution.^[^ ^6^ ^]^

Diverse photochemical approaches have been instrumental in controlling and understanding RNA structure and function. One such approach involves generating “cloaked” RNA that is only translated when chemical groups, globally incorporated into the RNA, are cleaved with UV light.^[^ ^7^ ^]^ Another approach involves incorporating synthetic, photocaged 5′ cap analogs known as FlashCaps into complex mRNAs to allow for photochemical triggering of mRNA translation upon UV light irradiation.^[^ ^8^ ^]^ This approach enables specific incorporation of a photocage to the cap and generates an unmodified RNA upon uncaging and requires extensive synthetic chemistry to produce caged cap analogs. In a related biochemical approach, chemoenzymatic modification of RNAs has also enabled the preparation and use of modified RNAs for photochemical control over gene expression.^[^ ^9^ ^]^ Alternatively, a thermoreversible reaction of nucleic acids with methylglyoxal can provide control over nucleic acid function, though use of this approach is limited by the need for specific incubation conditions and relatively high temperature.^[^ ^10^ ^]^ Another approach entails the construction of riboswitch‐based biosensors by integrating fluorogenic aptamers, enabling the acquisition of spatiotemporal information on target analytes within living cells, without necessitating labeling or invasive methodologies.^[^ ^11^ ^]^ Photochemistry has also been used to control gene expression by using light activated miRNAs, which enables manipulation of miRNA function and networks using UV irradiation with exceptional precision in both space and time.^[^ ^12^ ^]^ Indeed, the development of caged antagomir and caged miRNA has enabled light‐induced reversible miRNA activity in live zebrafish embryos.^[^ ^13^ ^]^ In addition, photocaged nucleobases that were site‐specifically incorporated into RNA duplexes, aided in developing light‐activated siRNA reagents.^[^ ^14^ ^]^ Light‐mediated regulation of RNA through photoprotecting groups (PPGs) allows reversible blocking and activation of RNA structure and interactions, thereby controlling RNA function.^[^ ^15^ ^]^ Although not photochemical, pharmacological repression of translation in live cells has also been accomplished by incorporating evolved aptamers for small molecules into mRNAs.^[^ ^16^ ^]^

**Figure 1:**
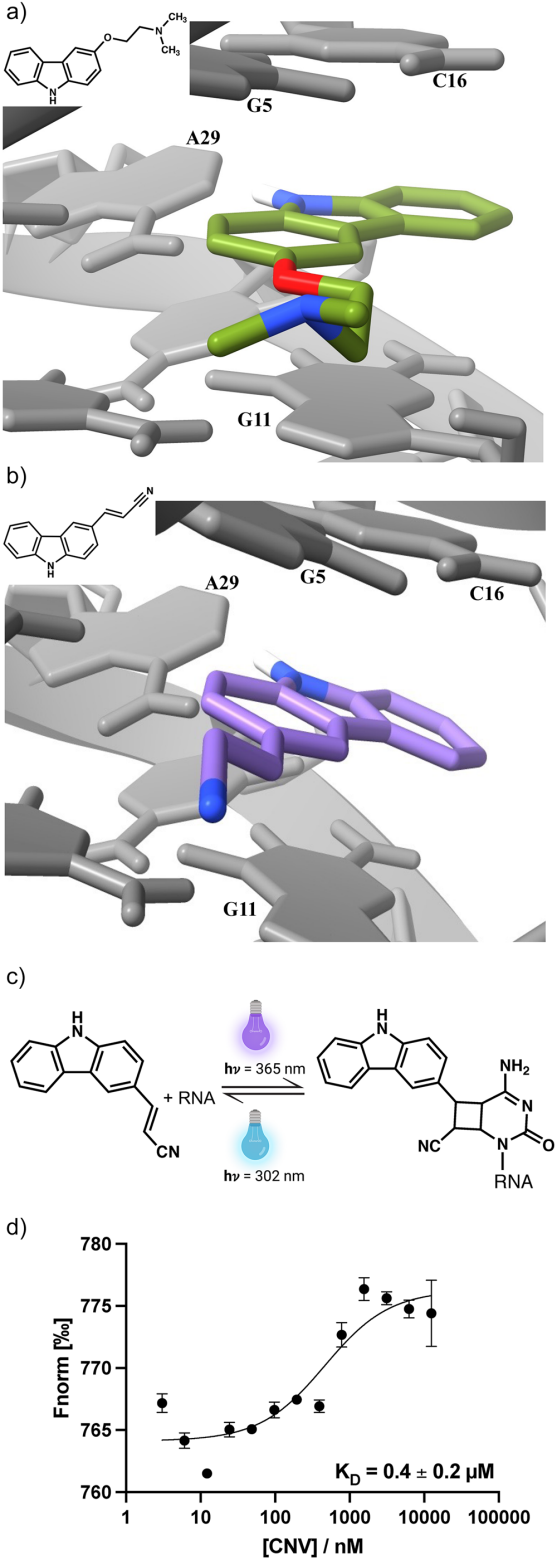
Ligand binding to PreQ_1_ riboswitch aptamers. a) X‐Ray co‐crystal structure of a carbazole‐containing ligand (PDB:6E1V) and b) docked structure of the CNV ligand adopting a similar binding pose. Images are generated using chimera.^[^ ^28^ ^]^ c) Reversible photocrosslinking of CNV probe to a pyrimidine base. d) Affinity measurement of CNV ligand with *Tte* PreQ_1_ riboswitch aptamer using microscale thermophoresis (MST). Error bars represent the standard deviation from three independent replicates.

Photochemical approaches have been used to probe RNA structure as well. Reagents, such as nicotinyl azide, have been developed to map solvent accessibility of purine bases through photochemical generation of nitrenes.^[^ ^17^ ^]^ Psoralen Analysis of RNA Interactions and structures (PARIS) has been applied to globally map duplex structures throughout the transcriptome.^[^ ^18^ ^]^ The PARIS approach leverages the ability of psoralen to non‐specifically intercalate and reversibly crosslink to duplex RNA structures, reading out RNA‐RNA basepairing interactions. Werewolf‐1 represents the inaugural instance of a synthetic riboswitch, demonstrating selective affinity for a singular photoisoform of its ligand, thereby effecting reversible control over bacterial RNA expression.^[^ ^19^ ^]^ The carbazole‐based cyanovinyl carbazole (CNV) molecule is another probe that can modify pyrimidine bases under 365 nm UV light, resulting in intrastrand crosslinking via a [2 + 2] cycloaddition.^[^ ^20^, ^21^, ^22^ ^]^ In this system, crosslinking can also be reversed when adducts are exposed to 302 nm UV irradiation, releasing an unmodified pyrimidine base and regenerating the CNV scaffold. To date, this probe has been used for interstrand crosslinking of double‐stranded nucleic acids when synthetically conjugated into the oligonucleotide chain via solid phase synthesis.^[^ ^23^ ^]^ With sequence‐specific CNV‐modified antisense oligonucleotides (AS‐ODN) and photocrosslinkable antisense probes (pcASOs), GFP gene expression has also been regulated via rapid photocrosslinking to complementary RNA.^[^ ^24^, ^25^ ^]^ Previously, our laboratory demonstrated that carbazole‐containing small molecule ligands similar to the CNV scaffold interact with PreQ_1_ riboswitch aptamers,^[^ ^26^ ^]^ distinct from other synthetic ligands for PreQ_1_ ligands.^[^ ^27^ ^]^ Despite having reasonably tight binding interactions, these compounds were inactive in single round transcriptional termination assays measuring switching activity. Still, access to an X‐ray co‐crystal structure demonstrated a molecular basis for recognition. Inspection of the crystal structure revealed that certain positions in the carbazole ring were likely to accommodate chemical substituents without compromising binding (Figure 1).

In this work, we report for the first time that small molecule probes based on the CNV ligand can be used to photochemically control the translation of complex mRNAs that encode wild‐type proteins by specifically and reversibly crosslinking to PreQ_1_ aptamers. This system relies exclusively on molecular recognition between a small molecule and RNA and enables reversible, covalent, light‐triggered modification of the RNA by a small molecule. We demonstrate that the CNV probe specifically and covalently modifies PreQ_1_ aptamers using multiple biochemical and biophysical techniques. Importantly, both crosslinking and uncaging are highly selective and specifically dependent on both the chemical structure of the probe as well as the RNA sequence/structure. Synthesis of a biotinylated CNV probe facilitated the generation of a homogeneously modified (“caged”) RNA population. The specificity of CNV probe was demonstrated by enriching the *Staphylococcus saprophyticus* (*Ssa*) aptamer sequence from human transcriptomes followed by RNA sequencing. Here, differential expression analysis revealed remarkably selective enrichment of the *Ssa* aptamer. This probe was utilized to generate and isolate uniformly caged ∼900 nt mRNA containing a PreQ_1_ aptamer in the 5′ UTR and a eGFP coding sequence. In both in vitro translation assays and when transfected into cells, the caged mRNA displayed repressed or no translation. However, upon irradiation with 302 nm light, the photocage was cleaved and translation was restored to levels equivalent to an unmodified mRNA. The regulation of GFP expression was quantified using flow cytometry and a cell line constitutively expressing mCherry served as internal control. To demonstrate the feasibility of this approach for controlling wild‐type protein expression, experiments were also performed on the mRNA encoding p53, a tumor suppressor, modified to incorporate a PreQ_1_ aptamer in the 5′UTR. Here, crosslinked mRNA did not express p53 until cells were irradiated at 302 nm. Thus, mRNAs containing the PreQ_1_ aptamer‐CNV probe ligand pair can be used to enable direct, precise photochemical activation of mRNA translation in live cells.

## Results and Discussion

### CNV Probe Binds to Preq_1_ RNA Aptamer

To visualize that the CNV ligand binds to the PreQ_1_ riboswitch, we performed docking simulations using ICM molsoft (Version 3.9–3a). A co‐crystal structure of *Thermoanaerobacter tengcongensis (Tte)*‐PreQ_1_ riboswitch^[^ ^29^, ^30^ ^]^ with the carbazole ligand (PDB:6E1 V)^[^ ^26^ ^]^ (Figure 1a) was used and the ligand was replaced by CNV in silico to obtain putative binding interactions. This revealed that like the carbazole ligand, CNV also could be occupying PreQ_1_ binding site and have a similar binding confirmation (Figure 1b). To demonstrate that the CNV ligand binds to PreQ_1_ aptamers, fluorescence titration was performed using microscale thermophoresis (MST). The fluorescence was measured at different temperatures and plotted as a function of ligand concentration. By fitting the dose response curve to a single site binding model, an equilibrium dissociation constant (*K*_D_) of 0.4 ± 0.2 µM was obtained (Figure 1d). Thus, the CNV ligand binds to the *Tte* PreQ_1_ aptamer with similar affinity to previously reported synthetic ligands.^[^ ^26^ ^]^

### CNV Probe Photocrosslinks to PreQ_1_ RNA Aptamer

**Figure 2:**
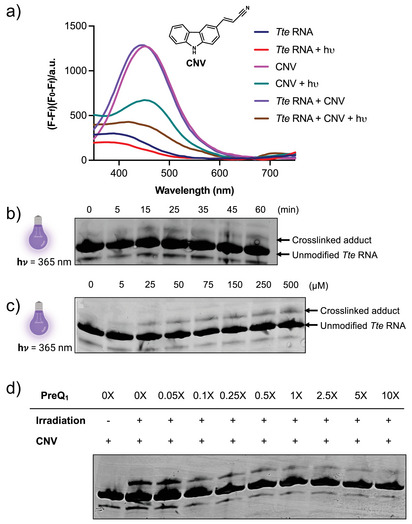
CNV probe specifically photocrosslinks to PreQ_1_ aptamer. a) CNV ligand fluorescence assays, indicating that photocrosslinking disrupts ligand fluorescence (Excitation wavelength: 330 nm) b) Gel shift assays illustrating formation of crosslinked adduct as a function of time (0–60 min) and c) dose (0–500 µM). d) PAGE assay showing that the CNV probe is biochemically competitive with PreQ_1_ ligand (0X–10X). Gels indicate representative images from triplicate assays.

To demonstrate crosslinking of CNV to the *Tte* PreQ_1_ aptamer, both mass spectrometry and spectroscopic assays were performed. Photocrosslinking assays were performed by folding *Tte* PreQ_1_ RNA in PreQ_1_ folding buffer, pre‐incubating with 75 µM CNV for 15 min, and irradiating under 365 nm UV light for 35 min. Fluorescence intensity was monitored after irradiation at 330 nm (the observed excitation maximum of CNV). Because crosslinking disrupts the chromophore in the CNV ligand, a decrease in fluorescence was observed (Figure 2a). In parallel, we performed a time course analysis of photocrosslinking with 75 µM CNV probe crosslinked to 50 µM *Tte* PreQ_1_ RNA by UV irradiation at 365 nm for 0 through 60 min and 15% of crosslinking was achieved at 35 min with no significant change with further increase in the time of crosslinking (Figure 2b). Subsequently, dose‐dependent photocrosslinking was performed at different concentrations of CNV ranging from 0 µM to 500 µM with 50 µM *Tte* PreQ_1_ aptamer. Crosslinked adducts were analyzed by denaturing PAGE, where they appear as a slower migrating band relative to the uncrosslinked RNA. It was observed that maximal adduct formation was achieved with 75 µM of CNV, which then remained constant with higher concentrations of probe (Figure 2c). In all samples, a faint lower band consistently appeared on the gel. Since all the RNAs used in the study are commercially purchased, this lower band could be a byproduct of RNA synthesis or labeling but does not appear to influence photocrosslinking or cleavage.

### PreQ_1_ Ligand Prevents Covalent Photocrosslinking by CNV

We next performed photocrosslinking experiments in the presence of increasing concentrations of PreQ_1_. Because the PreQ_1_ ligand has both low nanomolar affinity for the RNA and high binding specificity, excess ligand was expected to compete for CNV binding and inhibit crosslinking. In a PAGE assay, we observed that addition of PreQ_1_ resulted in a dose‐dependent competitive inhibition of CNV crosslinking. Thus, crosslinking occurs at the cognate ligand binding site and is biochemically competitive with the cognate ligand (Figure 2d).

### Photocrosslinking Using CNV Probe is Reversible

**Figure 3:**
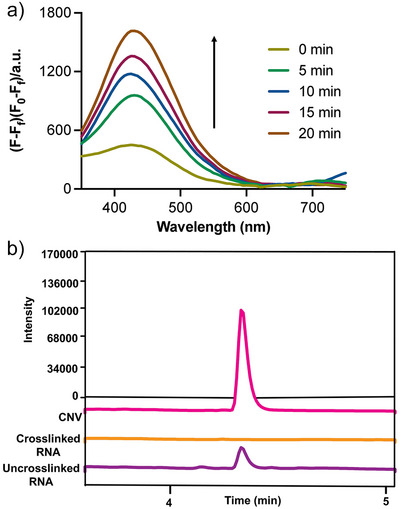
CNV reversibly photocrosslinks to PreQ_1_ aptamer. a) Time course of uncrosslinking upon irradiation at 302 nm showing enhanced fluorescence of CNV ligand with increase in time. b) LC/MS chromatograms showing mass of CNV ligand (pink), crosslinked RNA (orange), and observation of the CNV ligand (purple) after uncaging by exposure of RNA to 302 nm UV light for 10 min.

Having demonstrated that the CNV probe specifically and covalently crosslinks to the PreQ_1_ aptamer, we next investigated conditions to photochemically uncage the labeled RNA. Crosslinked *Tte* PreQ_1_ riboswitch aptamer was exposed to 302 nm UV light, and cleavage of the CNV ligand was monitored spectroscopically by tracking CNV fluorescence (Figure 3a). As expected, cleavage of the CNV ligand restores the fluorophore, resulting in an increase in fluorescence emission at 425 nm. Fluorescence increased as a function of time, and complete uncrosslinking was observed after 20 min of irradiation. This observation was orthogonally confirmed by LC/MS. Figure 3b illustrates an LC/MS chromatogram of the CNV ligand. The ligand is absent in a sample of crosslinked RNA. However, upon irradiation of modified RNA with 302 nm light for 10 minutes, the ligand can be observed, confirming that uncaging conditions release unaltered CNV ligand from the RNA. Taken together, these results demonstrate that the CNV ligand reversibly crosslinks to the *Tte* PreQ_1_ aptamer.

### Diverse PreQ_1_ Aptamers Have Varied Photocrosslinking Efficiency to the CNV Probe

**Figure 4:**
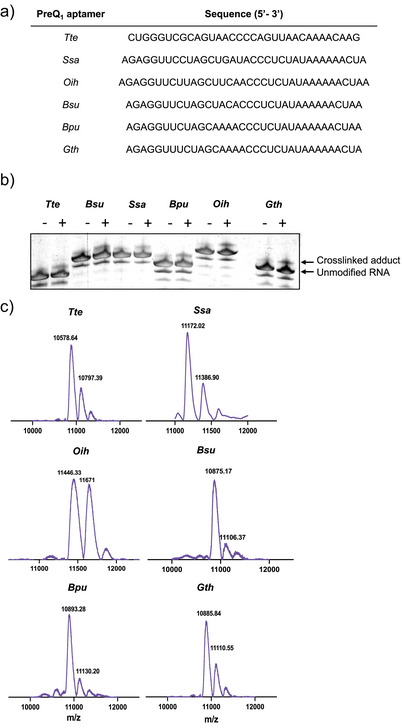
Effect of aptamer structure and RNA sequence on CNV crosslinking efficiency a) Sequence of PreQ_1_ aptamers from different species used in the study. b) Evaluation of photocrosslinking efficiency in different species of riboswitches by PAGE. Shown are representative gel‐shift images from three independent replicates. c) MALDI‐TOF mass spectrum of crosslinked aptamers in different riboswitches. Mass of native RNA and adduct are indicated above each peak. A higher molecular weight peak indicates formation of photocrosslinked adduct upon UV exposure.

Although initial experiments were performed with the *Tte* PreQ_1_ aptamer, multiple organisms have evolved PreQ_1_ riboswitches. Despite varying somewhat in sequence, these aptamers have largely conserved binding sites, suggesting that they could vary in crosslinking efficiency. We identified PreQ_1_ riboswitch aptamers from six species: *Tte*, *Bacillus subtilis (Bsu), Ssa, Oceanobacillus iheyensis (Oih), Bacillus pumilus (Bpu)*, and *Geobacillus thermodenitrificans (Gth)* ^[^ ^29^, ^30^, ^31^ ^]^ to evaluate photocrosslinking efficiency using the CNV ligand (Figure 4a). Each aptamer (50 µM) was irradiated for 35 min at 365 nm at RT in the presence of 75 µM CNV ligand, and crosslinking was monitored by gel shift on PAGE. All aptamers showed some evidence of adduct formation (Figure 4b). In parallel, MALDI‐TOF mass spectrometry was performed to confirm the identity of photocrosslinked adducts by mass (Figure 4d). Together these results indicate that although the CNV ligand crosslinks to various aptamers, aptamer sequence/structure influences crosslinking efficiency even when aptamers bind the same cognate ligand.

### Riboswitch Photocrosslinking and Uncrosslinking Efficiency Varies with the Structure of Small Molecule

**Figure 5:**
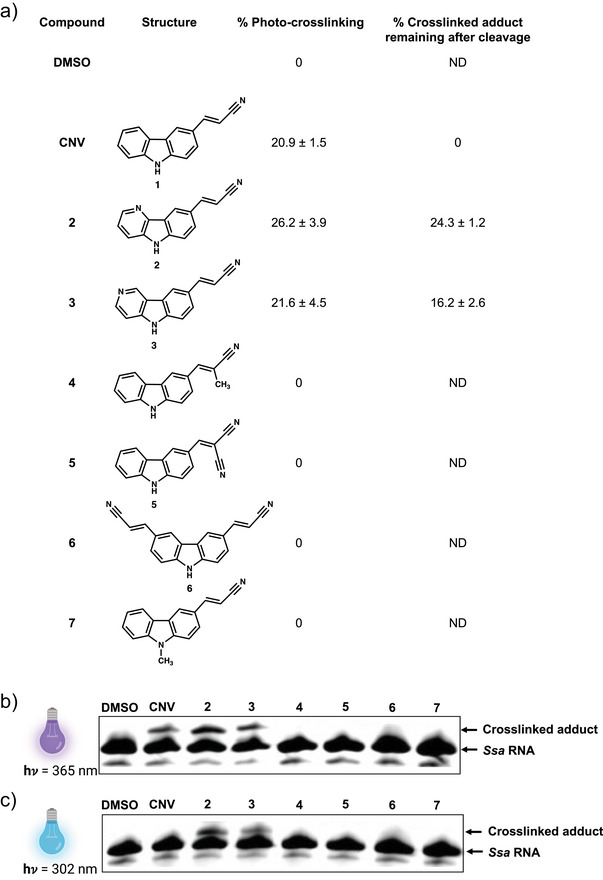
Diverse chemical structures have varying photocrosslinking/cleavage efficiency for the *Ssa*‐PreQ_1_ riboswitch aptamer. a) Table showing photocrosslinking and un‐photocrosslinking efficiencies of different compounds on *Ssa‐*PreQ_1_ RNA. Error bars indicate standard deviation, *n* = 3. ND=not determined. b) Gel analysis depicting photochemical crosslinking and c) uncrosslinking with different chemical analogs. Shown are representative images from three replicates.

Having demonstrated that aptamer sequence/structure influences crosslinking efficiency, we next evaluated the effect of the CNV ligand structure on crosslinking. We designed and synthesized seven analogs altering the structure of the CNV ligand to investigate the effects of ligand structure on crosslinking and uncrosslinking (Figure 5a). We evaluated the ability of each compound to photocrosslink to the *Ssa‐*PreQ_1_ aptamer. 50 µM of *Ssa‐*PreQ_1_ RNA was treated with 75 µM of each of the analogs and irradiated with 365 nm UV light for 35 min. Crosslinking adducts were observed by gel shift in PAGE. Out of these, CNV, **2**, and **3** showed evidence of crosslinked products, while **4**, **5,** and **6** did not. Notably, compound **7**, which differs from CNV solely by the presence of a methyl group on the carbazole nitrogen, showed no evidence of crosslinking, highlighting the specificity of recognition (Figure 5c). For uncaging, the CNV, **2**, and **3** adducts were irradiated with 302 nm light for 20 min and analyzed by PAGE. Only the CNV probe showed complete loss of crosslinked adduct, while **2** and **3** only showed minimal uncaging (Figure 5c).

### Enrichment of Homogenously Crosslinked Adducts

**Figure 6:**
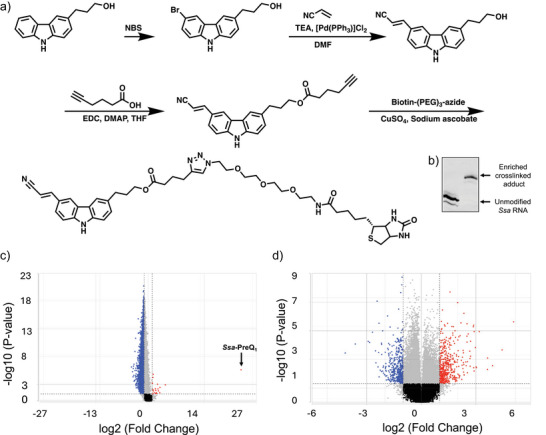
Enrichment of crosslinked aptamer. a) Chemical synthesis of a biotinylated CNV ligand for photocrosslinking and biochemical enrichment b) Gel image of the biotin‐enriched photocrosslinked aptamer after streptavidin‐mediated pull‐down. c) Volcano plot of *Ssa*‐PreQ_1_ aptamer enrichment using bio‐CNV in human transcriptomes by RNA sequencing. The enriched aptamer is highlighted. d) Volcano plot of differential gene expression showing the effect of competitive inhibition of biotinylated CNV probe by natural ligand. RNA sequencing analysis was completed with triplicate samples for each condition.

Due to the incomplete formation of crosslinked adducts in vitro, we recognized the need to obtain uniformly modified samples of RNA to perform further validation and also to rule out any effects by unmodified RNA in functional assays. To generate a homogenously labeled sample of crosslinked aptamer, chemical enrichment with a modified biotinylated CNV probe (bio‐CNV) was performed (Figure 6). We designed and chemically synthesized a CNV probe conjugated to a biotin handle for affinity enrichment using the synthetic route in Figure 6a. This probe‐based enrichment enabled us to interrogate the effects the modified RNA on a biological system. 50 µM *Ssa‐*PreQ_1_ RNA was photocrosslinked with 75 µM bio‐CNV probe for 35 min, and enrichment of crosslinked adducts was performed utilizing streptavidin beads. After photocrosslinking and enrichment/purification, a homogenously labeled sample of crosslinked PreQ_1_ aptamer was obtained (Figure 6b). The enrichment was also performed in human MCF7 cell line to assess the selectivity of CNV crosslinking in complex mixtures of RNAs. *Ssa*‐PreQ_1_ aptamer sequence was added to total RNA isolated from MCF7 cells. Next, bio‐CNV was added, and the sample was irradiated. A pull down was performed using streptavidin beads, and the RNA was analyzed for enrichment using RNAseq (Figure 6c). Here, the PreQ_1_ aptamer was the only RNA enriched, confirming that when the aptamer is present crosslinking is highly selective. In parallel, the same pulldown was performed in the presence of the cognate ligand PreQ_1_. These samples showed no enrichment of the *Ssa*‐PreQ_1_ aptamer, confirming the specificity of the interaction (Figure 6d).

### Harnessing Crosslinking to Control mRNA Translation

**Figure 7:**
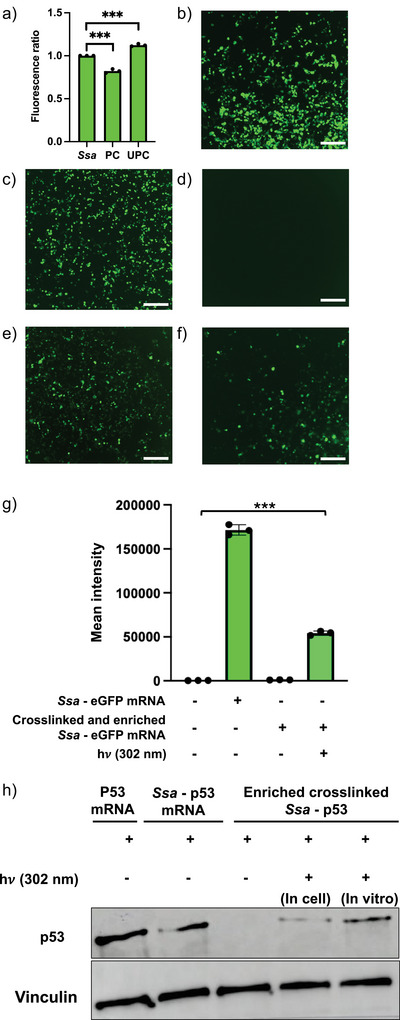
Reversible photocrosslinking enables photochemically controlled translation a) Relative fluorescence of unmodified (*Ssa*), bio‐CNV crosslinked (PC), and uncrosslinked (UPC) *Ssa*‐PreQ_1_ eGFP mRNA using an in vitro translation assay. Error bars indicate standard deviation of independent triplicate repeats. Each data point represents an individual replicate. Asterisks indicate *p* < 0.001. Images of HEK293T cells transfected with b) eGFP mRNA, c) *Ssa‐*PreQ_1_ eGFP mRNA, d) bio‐CNV photocrosslinked *Ssa‐*PreQ_1_‐eGFP mRNA, e) *Ssa*PreQ_1_‐eGFP mRNA uncrosslinked in vitro and transfected, and f) *Ssa*‐PreQ_1_‐eGFP mRNA uncrosslinked in live cells. Scale bars, 300 µm. Images are representative from three independent replicates. g) Mean intensities of eGFP fluorescence observed by flow cytometry in cells transfected with *Ssa*‐PreQ_1_‐eGFP mRNA, enriched crosslinked and uncrosslinked *Ssa*‐PreQ_1_‐eGFP mRNA. h) Western blot images showing expression of p53 with transfection of p53 mRNA only, *Ssa*‐PreQ_1_‐p53 mRNA, crosslinked and enriched *Ssa*‐PreQ_1_‐p53 mRNA and uncrosslinked conditions performed in both in mCherry‐expressing HEK 293T cells and in vitro. Gels are representative images from triplicate experiments.

Structure in RNA is known to impact translation, and covalent lesions on RNA would be expected to have similar effects. To demonstrate that reversible crosslinking can mediate conditional control over mRNA function, we ligated *Ssa*‐PreQ_1_ riboswitch aptamer to the 5′ end of an eGFP mRNA. For in vitro translation assays, the fluorescence intensity of eGFP protein was observed at 488 nm using a plate reader. These results indicated that crosslinked *Ssa‐*PreQ_1_‐eGFP RNA demonstrated a statistically significant but incomplete reduction in fluorescence intensity when compared to an unmodified eGFP mRNA. However, upon irradiation with 302 nm light for 20 min, *Ssa‐*PreQ_1_‐eGFP RNA was restored to slightly above wild‐type levels (Figure 7a). Notably, it is not unprecedented to observe slightly increased fluorescence (above the unreacted sample) upon uncaging modified RNAs.^[^ ^17^ ^]^

Next, these RNA constructs were transfected individually into HEK293T cells and imaged. Cells transfected with eGFP mRNA alone and unmodified *Ssa‐*PreQ_1_‐eGFP mRNA showed high levels of green fluorescent protein (Figure 7b,c). In contrast, crosslinked *Ssa*‐PreQ_1_‐eGFP mRNA exhibited no fluorescence, demonstrating that the crosslinked adduct inhibits proper translation in cells (Figure 7d). Uncaging the crosslinked mRNA before transfection resulted in near wild‐type levels of fluorescence (Figure 7e). Gratifyingly, uncaging could also be performed in live cells. Here, HEK293T cells were transfected with photocrosslinked *Ssa*‐PreQ_1_‐eGFP mRNA and irradiated with 302 nm UV light, after which a clear increase in eGFP fluorescence was observed. These results demonstrate that mRNA expression could be photochemically triggered using CNV‐based reversible photocrosslinking in cells. To illlustrate that the expression of eGFP RNA was specifically controlled by the CNV probe‐based crosslinking, HEK293T cells stably expressing mCherry were used. A flow‐cytometry based readout of the expression of eGFP was performed under several conditions (Figure 7g). Analysis of mCherry expression revealed no effect on protein levels under any condition (Figure S3). However, eGFP expression was highly repressed by the CNV‐ligand mediated crosslinking at 365 nm and can be restored after uncrosslinking with 302 nm light (Figure S3). The quantification of fluorescence intensity demonstrates that the crosslinked *Ssa*‐PreQ_1_‐eGFP mRNA resulted into complete suppression of eGFP expression. When the RNA was uncrosslinked both in vitro (before transfection) and by irradiating live cells (after transfection), the expression of eGFP was dramatically restored (Figure 7g).

Finally, to demonstrate that the CNV probe can be used to control the expression of a wild‐type protein of biological relevance, we generated a modified mRNA that encodes for p53, a tumor suppressor (Figure 7h). We engineered an *Ssa* PreQ_1_ aptamer sequence in the 5′ UTR, upstream of the p53 coding sequence, to express a wild‐type protein (*Ssa‐*PreQ_1_‐p53). Cells were transfected with bio‐CNV crosslinked *Ssa‐*PreQ_1_‐p53 (enriched to homogeneity) and *Ssa‐*PreQ_1_‐p53 that was uncrosslinked prior to transfection. Uncrosslinking was also performed in live cells and protein expression was assessed under each condition. Western blots illustrate no expression of p53 upon transfection with bio‐CNV crosslinked *Ssa‐*PreQ_1_‐p53. However, cells transfected with *Ssa‐*PreQ_1_‐p53 uncrosslinked in vitro before transfection showed high levels of p53, indicating that uncrosslinking does not prevent translation. p53 could also be expressed by uncrosslinking in live cells (Figure 7h). Together, these experiments illustrate that both model (eGFP) and wild‐type (p53) proteins can be conditionally, photochemically expressed in cells using the CNV‐PreQ_1_ aptamer system.

## Conclusion

Exploitation of chemical probes provides unique opportunities to control the function of complex RNAs. Moreover, precise control of RNA biology using chemical approaches and light‐mediated techniques has opened new avenues for the potential development of RNA‐based therapeutics and the study of complex biological systems. To date, chemical approaches to control RNA have relied on global chemical modification, chemoenzymatic modification, or chemical synthesis. By comparison, the interaction and specific modification of RNA with photoreactive ligands relying exclusively on molecular recognition offers a complementary means of precisely activating translation and gene expression that is efficient and does not require any additional components. By utilizing molecular recognition and reversible crosslinking of a small molecule with an RNA aptamer, it is possible to achieve specific incorporation and traceless cleavage of a small molecule tag from a complex RNA and achieving conditional control over gene expression.

This work illustrates the capacity of the CNV ligand to selectively recognize the PreQ_1_ RNA aptamer structure and control RNA function through reversible, photochemically triggered covalent interactions. This effect is specific to both the sequence/structure of the aptamer as well as the CNV probe itself. Incorporation of this system into a messenger RNA encoding eGFP demonstrated that the crosslinked aptamer within the 5′ UTR suppressed translation, while uncaging with UV light restored the ability of the modified RNA to be translated both in vitro and in live cells. Furthermore, the biological significance and applicability of the system were demonstrated by achieving precise and controlled expression of a native protein, p53, via reversible crosslinking facilitated by the CNV probe. Importantly, because this approach only requires modification of the 5′ UTR, it enables conditional expression of unmodified, wild‐type proteins of interest. Thus, photochemically triggered translation offers precise temporal regulation of gene expression, of both model and wild‐type proteins, in an approach that exclusively employs molecular recognition and UV light. The approach outlined here is straightforward and applicable to a wide range of complex RNAs and enables photochemical manipulation of complex RNA function. As a result, this technique holds considerable promise as an approach for regulating RNA function via photochemical means in diverse biological contexts.

## Conflict of Interests

The authors declare no conflict of interest.

## Supporting information



Supporting Information

## Data Availability

The RNA sequencing data for enrichment analysis were deposited to the NCBI, https://www.ncbi.nlm.nih.gov/bioproject (BioProject ID PRJNA 1113586).
