# Supplementary material for: Harnessing Molecular Recognition for Small‐Molecule‐Mediated Reversible Photochemical Control Over mRNA Translation
Source: Angew Chem Int Ed Engl. 2025 Apr 22;64(22):e202503078. doi: 10.1002/anie.202503078 (PMC12105704; doi:10.1002/anie.202503078)
Supplement: Supplementary file 1 — Supporting Information [file ANIE-64-e202503078-s001.pdf]

Harnessing molecular recognition for small molecule-mediated, reversible photochemical control over mRNA translation

Supporting Information

Shaifaly Parmar[#1], Logan Tenney[#1], Xiao Liang[1], John T. Routzahn IV[1], Christopher D. Sibley[1], John S. Schneekloth Jr.[1*]

[1] Chemical Biology Laboratory, Center for Cancer Research, National Cancer Institute, Frederick, MD 21702-1201, USA.

*e-mail: schneeklothjs@mail.nih.gov

Experimental Procedures

Microscale thermophoresis

Microscale thermophoresis (MST) assay was performed using cy5-labelled *Tte* $PreQ_1$ RNA (5'-CUGGGUCGCAGUAACCCCAGUUAACAAAACAAG-3', Horizon Discovery). The RNA was diluted into 1X $PreQ_1$ folding buffer (50 mM Tris, 100 mM KCl, 1 mM $MgCl_2$, pH 7.5) to a concentration of 100 nM and was annealed by heating to 75 °C for 5 minutes, followed by slowly cooling to room temperature for 30 minutes. Serial dilutions with 100 µM CNV solution was prepared in 10% DMSO in the 1X $PreQ_1$ buffer. RNA and CNV were mixed in equal quantities maintaining a total of 5% final DMSO concentration and were allowed to equilibrate for 15 minutes at room temperature. The samples were then photo-crosslinked at 365 nm for 35 minutes at RT. Using premium coated capillaries, MST was conducted in triplicate on a Monolith NT.115 system (NanoTemper Technologies). The dissociation constant (K_D) was determined by plotting ligand concentration and by using a single-site binding model.

Gel electrophoresis

Unlabeled $PreQ_1$ RNAs were diluted into 1X $PreQ_1$ buffer to a concentration of 100 µM (50 µM final concentration in solution) and annealed by heating to 75 °C for 5 minutes, followed by slowly cooling to room temperature for 30 minutes. For the dose-response, CNV was prepared in 10% DMSO in the buffer and added to the RNA solution to make the final concentration ranging from 0 to 500 µM, maintaining a 5% final DMSO concentration. After addition of CNV ligand, the

samples were allowed to equilibrate for 15 minutes at room temperature. The samples were then photo-crosslinked at 365 nm for 35 minutes. For the time-dependent analysis, 75 µM of CNV and 50 µM RNA were allowed to equilibrate for 15 minutes at room temperature and then photo-crosslinked at 365 nm for different time periods ranging from 0 through 60 minutes. For competitive crosslinking, to 50 µM RNA, 75 µM of CNV is added followed by increasing concentrations of $PreQ_1$ and allowed to equilibrate for 15 minutes at room temperature. They are photo-crosslinked at 365 nm for 35 minutes. 17% denaturing PAGE gel was run at 75 V for 3 h to separate the bands. The gel was then stained with SybrGold for 10 minutes and visualized using a Typhoon FLA 9500 (GE) or AMERSHAM Image Quant 800 (GE) or Image Quant LAS 4000 (GE).

LC-MS

Unlabeled *Tte*-$PreQ_1$ RNA was diluted into 1X $PreQ_1$ buffer to a concentration of 100 µM and annealed by heating to 75 °C for 5 minutes, followed by slowly cooling to room temperature for 30 minutes. 150 µM CNV solution was prepared in 10% DMSO in the 1X $PreQ_1$ buffer. 10 µl each of *Tte*-$PreQ_1$ RNA and CNV were mixed to make a final concentration of 50 µM and 75 µM respectively and allowed to incubate for 15 minutes at room temperature. The mixture is subjected to 365 nm UV light for 35 minutes for crosslinking. To uncrosslink, the crosslinked mixture is subjected to 302 nm UV light for 20 minutes. No crosslinking, crosslinked and uncrosslinked samples were analyzed on (ESI)-LC/MS on an LTQ-Orbitrap-XL LC/MS system.

(A)

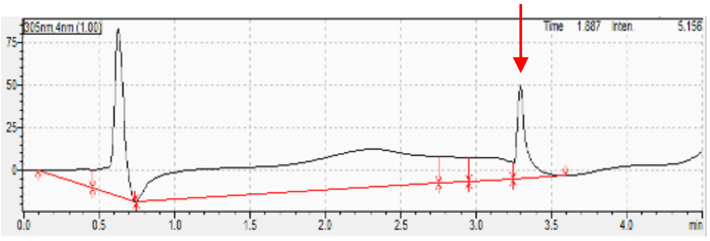

(B)

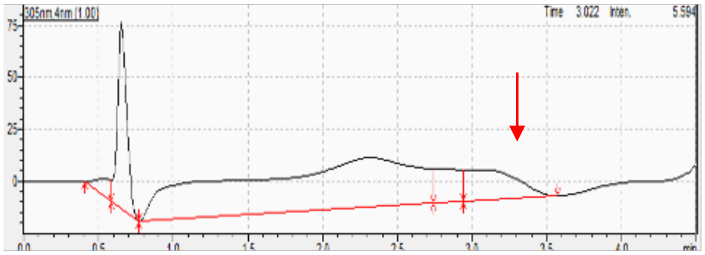

(C)

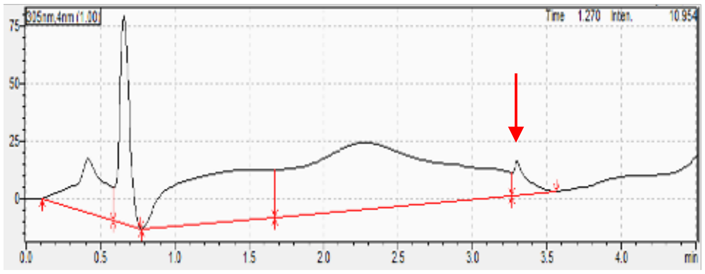

Figure S1: LC/MS chromatograms of (A) CNV ligand (B) CNV ligand photocrosslinked to RNA (C) CNV ligand after uncrosslinking. Red arrows denote peak of interest at 3.3 min.

MALDI-TOF

Six unlabeled $PreQ_1$ RNAs (IDT, Table 2) were diluted in 1X $PreQ_1$ buffer to a concentration of 100 µM and annealed by heating to 75 °C for 5 minutes, followed by slowly cooling to room temperature for 30 minutes. 150 µM CNV solution was prepared in 10% DMSO in the 1X $PreQ_1$ buffer. 10 µl each of $PreQ_1$ RNA and CNV were mixed to make a final concentration of 50 µM and 75 µM respectively and allowed to incubate for 15 minutes at room temperature. The mixture is subjected to 365 nm UV light for 35 minutes for crosslinking. The samples were then assessed for extent of crosslinking by negative ion MALDI operated in linear mode over a 5–20 kDa range.

(A) *Tte* $PreQ_1$

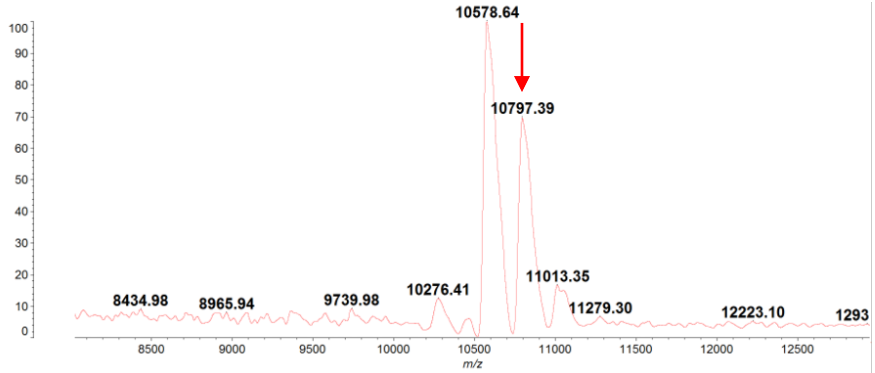

(B) *Ssa* $PreQ_1$

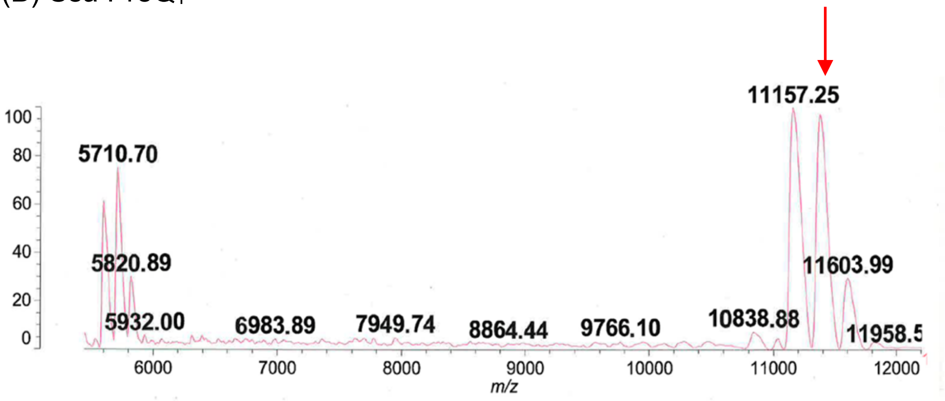

(C) *Oih* $PreQ_1$

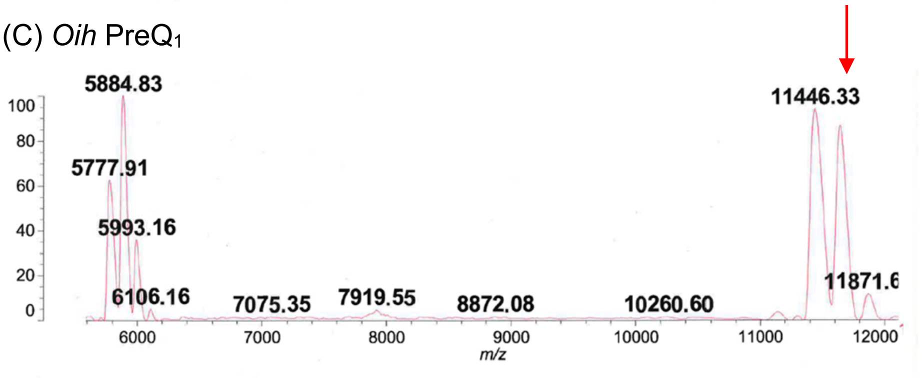

(D) *Bsu* $PreQ_1$

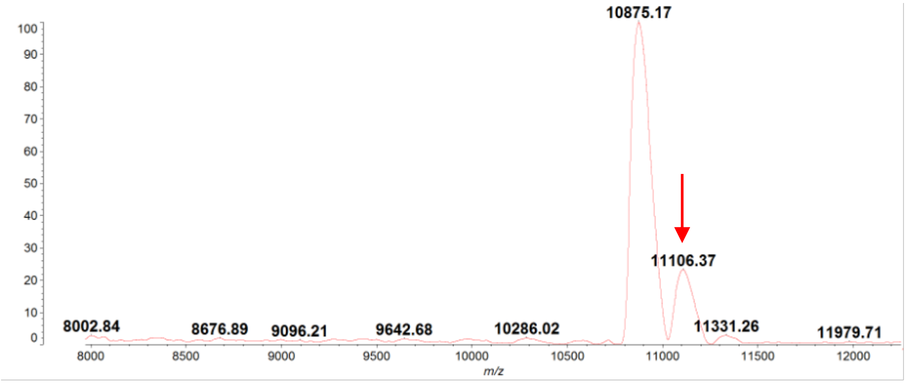

(E) *Bpu* $PreQ_1$

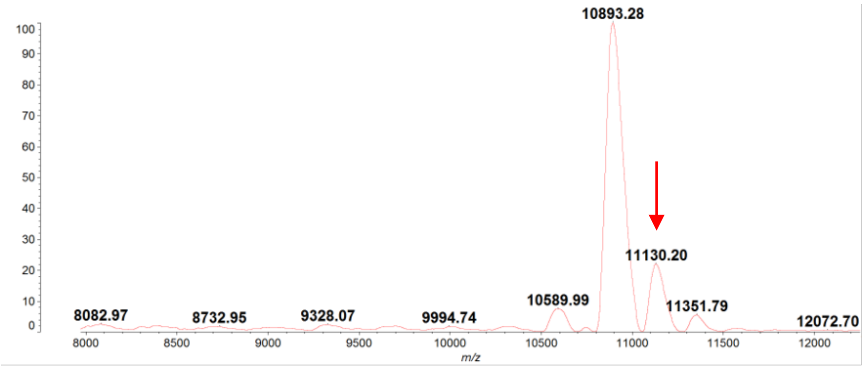

(F) *Gth* $PreQ_1$

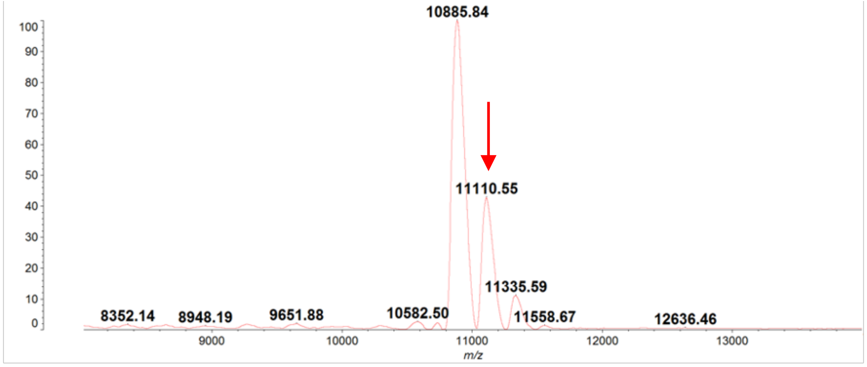

Figure S2: MALDI-TOF mass spectrum of different $PreQ_1$ riboswitches. Red arrows show the mass of crosslinked adducts.

Pull down of photo-crosslinked *Ssa*-$PreQ_1$ RNA using biotinylated CNV

Ssa-$PreQ_1$ RNA (IDT, Table 2) was folded using 1X $PreQ_1$ folding buffer and incubated at 75 °C for 5 minutes and cooled to room temperature for 30 minutes. The folded RNA was incubated with 75 μM of CNV-biotin for another 15 minutes at room temperature and then photo-crosslinked at 365 nm for 35 minutes. This photo-crosslinked RNA was purified by ethanol precipitation overnight and resuspended in water. Using streptavidin magnetic beads (NEB #S1420), biotinylated CNV probe crosslinked to RNA was pulled out following the manufacturer's protocol and eluted using a formamide elution buffer. The eluted RNA was checked on gel for integrity and quality and stored at -20 °C after purification by RNA clean and concentrator-5 (Zymo research).

RNA sequencing for enrichment analysis

MCF7 cells were maintained in RPMI-1640 media (Gibco) supplemented with 10% fetal bovine serum (FBS), 1% glutamine, and 1% puromycin in a 5% CO_2 humidified incubator at 37°C. Cells (~80% confluent) were harvested by trypsinization and suspended in 1X PBS. TRIzol (Invitrogen) was added to the cells and total RNA was isolated using manufacturer's instructions. DNase (NEB) digestion was performed following the manufacturer's protocol and purified using columns (Zymo RNA Clean & Concentrator-25). The treatment conditions include the addition of (A) 5% DMSO (control), (B) only 250 μM Biotinylated CNV probe, (C) 1 μM *Ssa*-$PreQ_1$ riboswitch aptamer for spike-in (folded in 1X $PreQ_1$ folding buffer as described above) and 250 μM Biotinylated CNV probe for pull down and (D) 1 μM spiked in *Ssa*-$PreQ_1$ riboswitch aptamer, 500 μM $PreQ_1$ ligand

(for competition) and pulled down by 250 µM Biotinylated CNV probe. The total RNA is treated in three independent replicates and crosslinked and enriched as described above. The samples were then sequenced on NextSeq 2000 P2 using paired-end sequencing. The samples had 37 to 66 million pass filter reads with more than 88% of bases above the quality score of Q30. Reads of the samples were trimmed for adapters and low-quality bases using Partek flow software was used for data analysis using standard data filters. Alignment was done building a reference that comprised of human genome (hg38) along with *Ssa* aptamer sequence using STAR.

Preparation of eGFP RNA by *in-vitro* transcription

pcDNA3-eGFP, a mammalian enhanced GFP expression vector was purchased (Addgene #13031) and linearized using *XbaI* enzyme (NEB). 5 µg linearized plasmid was used as the template for *in vitro* transcription using HiScribe™ T7 High Yield RNA Synthesis Kit (NEB) and was purified by LiCl precipitation. The eGFP RNA quality and integrity was checked on a bioanalyzer (2100 Bioanalyzer, Agilent).

Ligation, capping and poly-A tailing of the *Ssa*-PreQ$_1$-eGFP RNA

5 µg of the purified photo-crosslinked Ss-PreQ$_1$ RNA was ligated upstream to 5 µg eGFP-RNA using T4 RNA ligase (NEB) following manufacturer's instructions and purified using RNA clean and concentrator-25 (Zymo research). The ligated RNA was capped used vaccinia capping system (NEB) and was poly-A tailed used *E.coli* poly(A) polymerase (NEB) using manufacturer's protocol. The capped and tailed RNA was purified using RNA clean and concentrator (Zymo research). 1 µg of the purified photo-crosslinked *Ssa*-PreQ$_1$-eGFP RNA was subjected to un-photo-crosslinking by UV exposure of 302 nm for 10 minutes.

***In vitro* and *in cellulo* translation of *Ssa*-eGFP RNA**

The photo-crosslinked and un-photo-crosslinked *Ssa*-PreQ$_1$-eGFP RNA was used for *in vitro* translation using Rabbit Reticulocyte Lysate System (Promega) following manufacturer's protocol. Fluorescence intensity of the translated products were recorded using a microplate reader at emission of 485/20 nm and absorption at 530/25 nm with a gain of 125. The fluorescence intensities were normalized, and fold change was calculated with respect to no photo-crosslinking sample. For in-cell experiments, HEK293T cells were transfected with the eGFP RNA, crosslinked and uncrosslinked *Ssa*-PreQ$_1$-eGFP RNA constructs using Lipofectamine MessengerMAX (Invitrogen) using manufacturer's protocol. The cells were imaged on a Cytation 5 Cell Imaging Multimode Reader (Agilent).

Flow cytometry

HEK 293T-ACE2.TMPRSS2 (mCherry) cells were maintained in DMEM media (Gibco) supplemented with 10% fetal bovine serum (FBS), 1% glutamine, and 1% penicillin/streptomycin in a 5% CO_2 humidified incubator at 37°C. Cells were transfected with the eGFP RNA, crosslinked and un-crosslinked *Ssa*-PreQ$_1$-eGFP RNA constructs using Lipofectamine MessengerMAX (Invitrogen) using manufacturer's protocol. The cells were imaged on a Cytation 5 Cell Imaging Multimode Reader (Agilent). For flow cytometry, cells were harvested by trypsinization and suspended in 1X PBS. Cellular fluorescence was measured using FACSymphony and analyzed by FlowJo™ v10 Software.

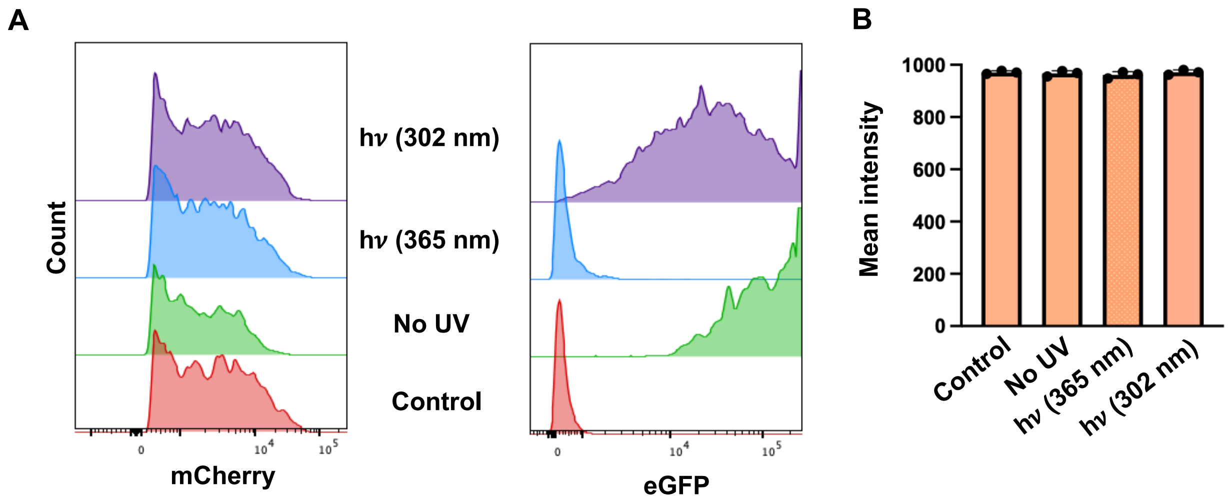

Figure S3: Flow cytometry histogram analysis of mCherry and eGFP expression in live cells. (A) The expression of mCherry and eGFP was performed in HEK 293T-ACE2.TMPRSS2 (mCherry) cells with no transfection (Control), *Ssa*-PreQ$_1$-eGFP mRNA with no crosslinking (No UV), crosslinked *Ssa*-PreQ$_1$-eGFP mRNA (hν, 365 nm), and uncrosslinked *Ssa*-PreQ$_1$-eGFP mRNA (hν, 302 nm). (B) Quantified mean fluorescence intensity of mCherry observed by flow cytometry.

Preparation of *Ssa*-PreQ$_1$-p53 RNA

pcDNA3.1(+)-C-DYK mammalian expression vector was engineered to contain the *Ssa*-PreQ$_1$ aptamer sequence followed by a p53 sequence (NM_001276760.2). This construct follows a T7 promoter sequence. Using this plasmid, *Ssa*-PreQ$_1$-p53 RNA was synthesized using standard protocols of HiScribe™ T7 High Yield RNA Synthesis Kit (NEB). The RNA was purified and quantified. This RNA was crosslinked and enriched using the methods described above using biotinylated CNV. The enriched RNA is also uncrosslinked *in vitro* as described above and these RNAs are used for transfection of HEK 293T cells.

Western blotting

HEK-293T cells were maintained in DMEM media (Gibco) supplemented with 10% fetal bovine serum (FBS), 1% glutamine, and 1% penicillin/streptomycin in a 5% CO_2 humidified incubator at 37°C. Cells were transfected with the control, crosslinked and uncrosslinked *Ssa*-PreQ$_1$-p53 RNA constructs using Lipofectamine MessengerMAX (Invitrogen) using manufacturer's protocol. After a wash with 1X PBS, cells were trypsinized and pelleted. 100ul Pierce RIPA lysis and extraction buffer (thermos scientific) with 1X protease inhibitor cocktail (Millipore sigma) was used to resuspend the pellet by vigorous pipetting and left at RT for 10 mins followed by high-speed centrifugation for 15 min. The supernatant was then transferred to a new vial and the protein was quantified using Qubit protein assay kit (Invitrogen). The samples were loaded on a pre-cast NuPAGE 4-12% Bis-tris gel (Invitrogen). Western blotting was followed using standard procedure and the antibodies used for the detection of proteins were, p53 (1:5000), Anti-rabbit IgG, HRP-linked Antibody (1:5000), Vinculin (E1E9V) XP® Rabbit mAb (HRP conjugate) (1:5000) (Cell signaling technology). Imaging of blots was performed on Amersham ImageQuant 800 instrument using SuperSignal West Atto Ultimate Sensitivity Chemiluminescent Substrate (Thermo scientific).

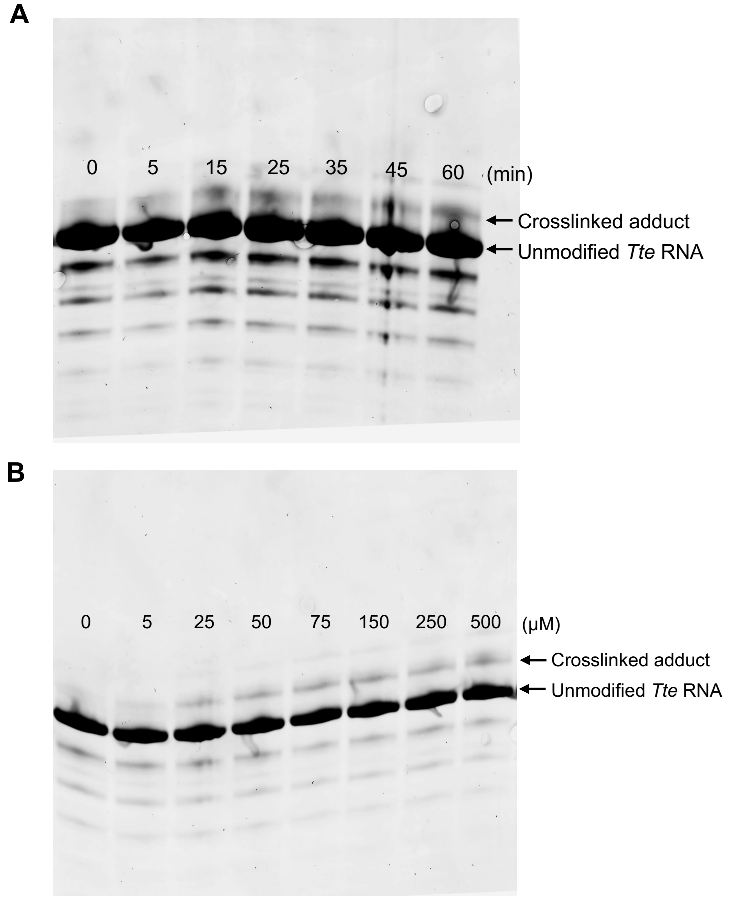

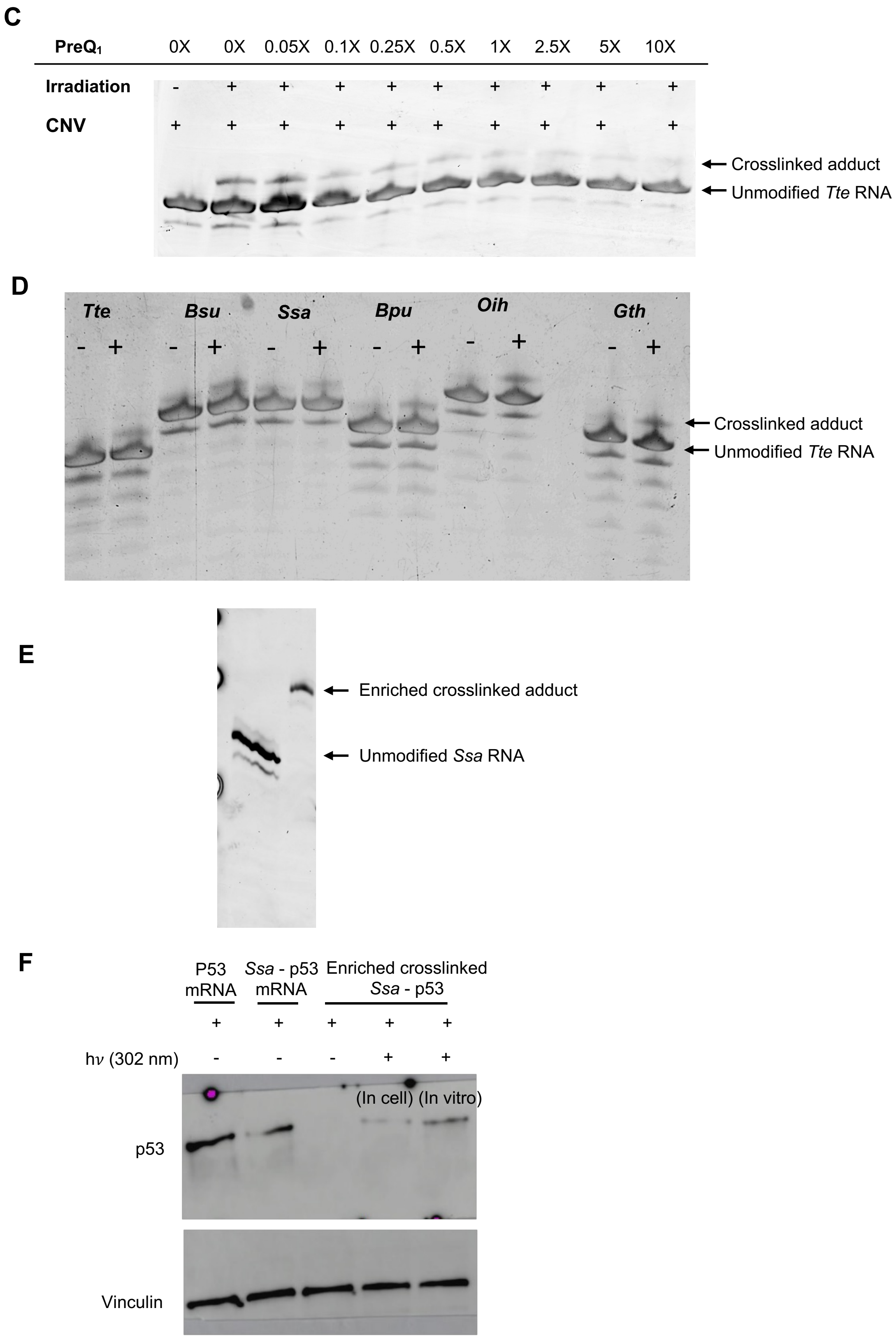
C
PreQ1
0X
0X
0.05X
0.1X
0.25X
0.5X
1X
2.5X
5X
10X
Irradiation
CNV
Crosslinked adduct
Unmodified Tte RNA
D
Tte
Bsu
Ssa
Bpu
Oih
Gth
Crosslinked adduct
Unmodified Tte RNA
E
Enriched crosslinked adduct
Unmodified Ssa RNA
F
P53 mRNA
Ssa - p53 mRNA
Enriched crosslinked Ssa - p53
hν (302 nm)
(In cell) (In vitro)
p53
Vinculin

Figure S4: Images of the denaturing PAGE gel analysis. (A) Time-based photocrosslinking of RNA with CNV probe, (B) photocrosslinking of RNA with increasing concentrations of CNV probe, (C) competitive photocrosslinking analysis of RNA in the presence of increasing concentrations of PreQ$_1$, (D) photocrosslinking of different PreQ$_1$ riboswitches with CNV probe, (E) photocrosslinked and enriched RNA adduct with bio-CNV probe. (F) Western blot showing p53 expression with transfection of p53 mRNA only, *Ssa*-PreQ$_1$-p53 mRNA, crosslinked and enriched *Ssa*-PreQ$_1$-p53 mRNA and uncrosslinked conditions in cells and in vitro.

General Chemistry Methods:

All chemical reagents were obtained from commercial suppliers and used without further purification. Solvents were removed using a Buchi rotary evaporator under reduced pressure. Flash column chromatography was performed using a Teledyne ISCO CombiFlash Rf automated chromatography system. ^{1}H and ^{13}C NMR spectra were recorded on a Bruker AVANCE III spectrometer at 500 MHz or at 125 MHz, respectively at 25 °C and are reported relative to deuterated solvent signals. Data for 1H NMR spectra are reported as follows: chemical shift (δ ppm), multiplicity (s = singlet, d = doublet, dd = doublet doublet, m = multiplet), coupling constants (Hz), and integration. Data for ^{13}C NMR spectra are reported in terms of chemical shift. All data was processed and visualized with MestReNova software (Version 14.1.1-24571). High resolution mass spectrometry (HRMS) data were acquired in positive ion mode on an Orbitrap LTQ-XL system (Thermo Fisher Scientific Inc.) that was configured with a UV-visible DAD detector in-line at 30K resolution by ESI.

Synthesis of CNV analogs

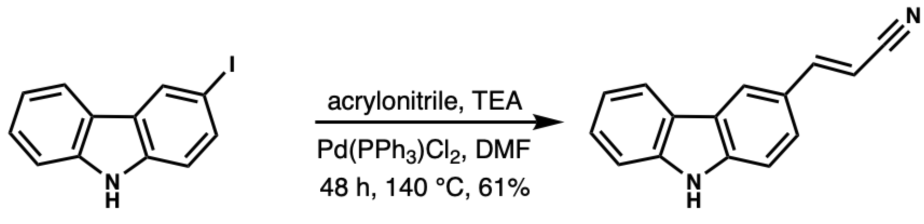

1

To a reaction flask was added 3-iodo-9H-carbazole (103.7 mg, 0.35 mmol) and acrylonitrile (37.5 mg, 2.48 mmol) in DMF (3 mL). TEA (178.4 mg, 1.75 mmol) was then added to the reaction mixture, followed by the addition of bis(triphenylphosphine)palladium (II) dichloride (25.3 mg, 0.036 mmol). The reaction was stirred at room temperature for 20 minutes and was then heated to 140°C and stirred for 48 h. The mixture was then extracted with EtOAc (8 mL x 3) and washed

with saturated $NaHCO_3$ and brine. The combined organic layers were dried over $MgSO_4$, filtered, and concentrated *in vacuo*. The resulting residue was purified by ISCO flash column chromatography (0-100% ethyl acetate in hexanes) to yield **1** (46.6 mg, 61%) as an off-white solid. ^{1}H NMR (500 MHz, DMSO-d_6): δ 11.60 (s, 1H), 8.45 (s, 1H), 8.04–8.01 (m, 2H), 7.17–7.14 (m, 1H), 7.12 (d, J = 9.9 Hz, 1H), 3.85 (s, 3H); ^{13}C NMR (125 MHz, DMSO-d_6): δ 151.8, 141.4, 140.3, 126.3, 125.3, 124.7, 122.7, 122.3, 120.9, 120.3, 119.7, 119.4, 111.5, 111.4, 92.3; HRMS: (ESI+) m/z calculated for $C_{15}H_{11}N_2$ [M+H]$^+$: 219.0917, found: 219.0909.

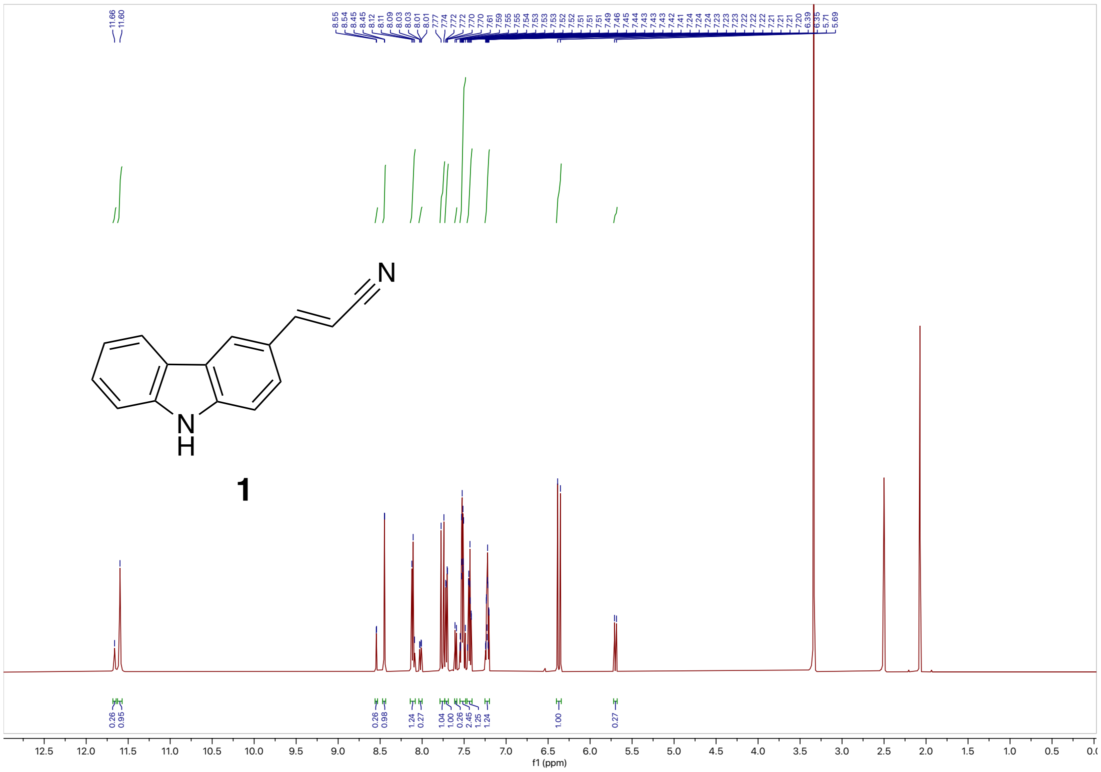

^{1}H NMR spectrum of **1** in DMSO-d_6 (500 Hz) at 25 °C.

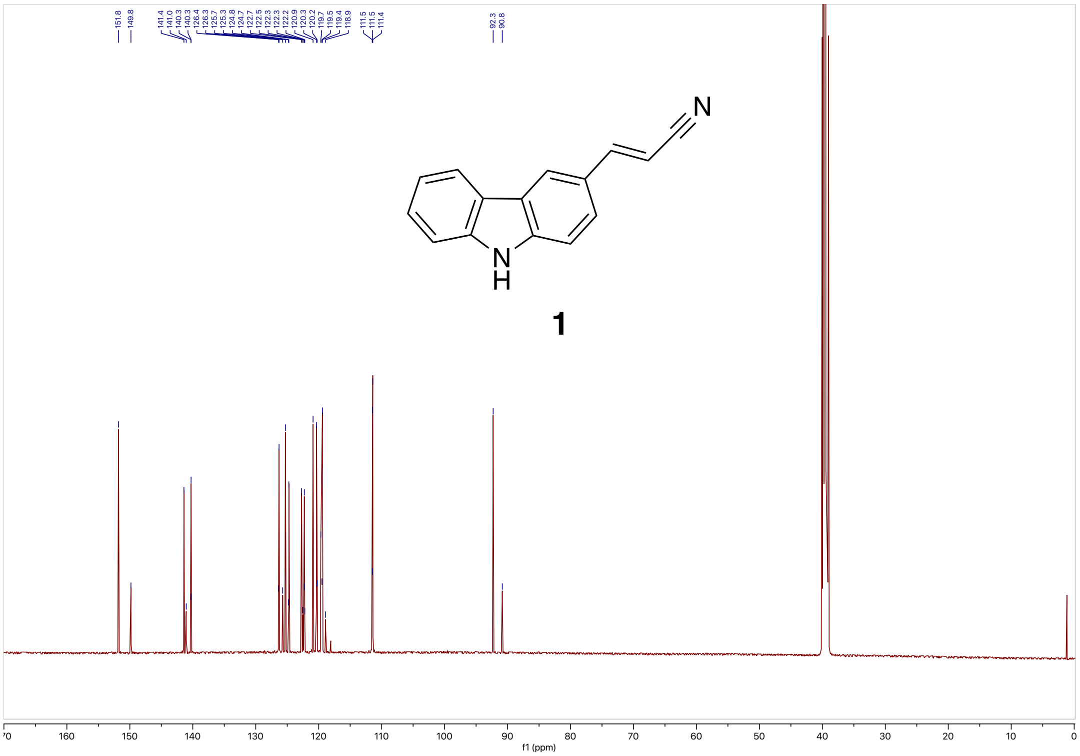

^{13}C NMR spectrum of **1** in DMSO-d_6 (125 Hz) at 25 °C.

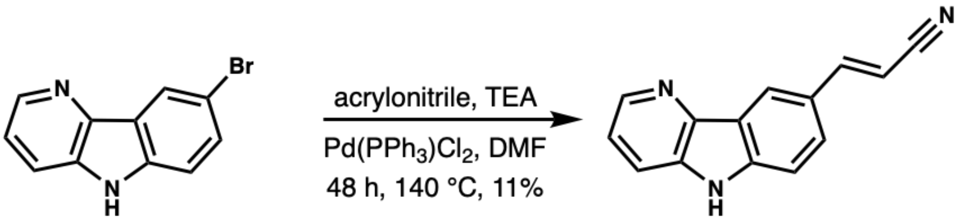

2

To a reaction flask was added 8-bromo-5*H*-pyrido[3,2-b]indole (93.7 mg, 0.38 mmol) and acrylonitrile (40.3 mg, 0.76 mmol) in DMF (3 mL). TEA (178.4 mg, 1.75 mmol) was then added to the reaction mixture, followed by the addition of bis(triphenylphosphine)palladium(II) dichloride (25.3 mg, 0.036 mmol). The reaction was stirred at room temperature for 20 minutes and was then heated to 140 °C and stirred for 48 h. The mixture was then extracted with EtOAc (8 mL x 3) and washed with saturated $NaHCO_3$ and brine. The combined organic layers were dried over $MgSO_4$, filtered and concentrated *in vacuo*. The resulting residue was purified by ISCO flash column chromatography (0-100% ethyl acetate in hexanes) to yield **2** (23.8 mg, 29%) as an off-white solid. ^{1}H NMR (500 MHz, DMSO-d_6): δ 11.76 (s, 1H), 8.51–8.49 (m, 2H), 7.93 (dd, *J* = 8.2, 1.2 Hz, 1H), 7.86–7.83 (m, 2H), 7.60 (d, *J* = 8.6 Hz, 1H), 7.43 (dd, *J* = 8.2, 4.6 Hz, 1H), 6.46 (d, *J* = 6.8 Hz, 1H); ^{13}C NMR (125 MHz, DMSO-d_6): δ 151.5, 142.0, 141.9, 141.0, 133.6, 126.5, 125.6,

121.8, 121.0, 120.8, 119.6, 118.7, 112.3, 93.3; HRMS: (ESI+) m/z calculated for $C_{14}H_{10}N_3$ [M+H]$^+$: 220.0869, found: 220.0863.

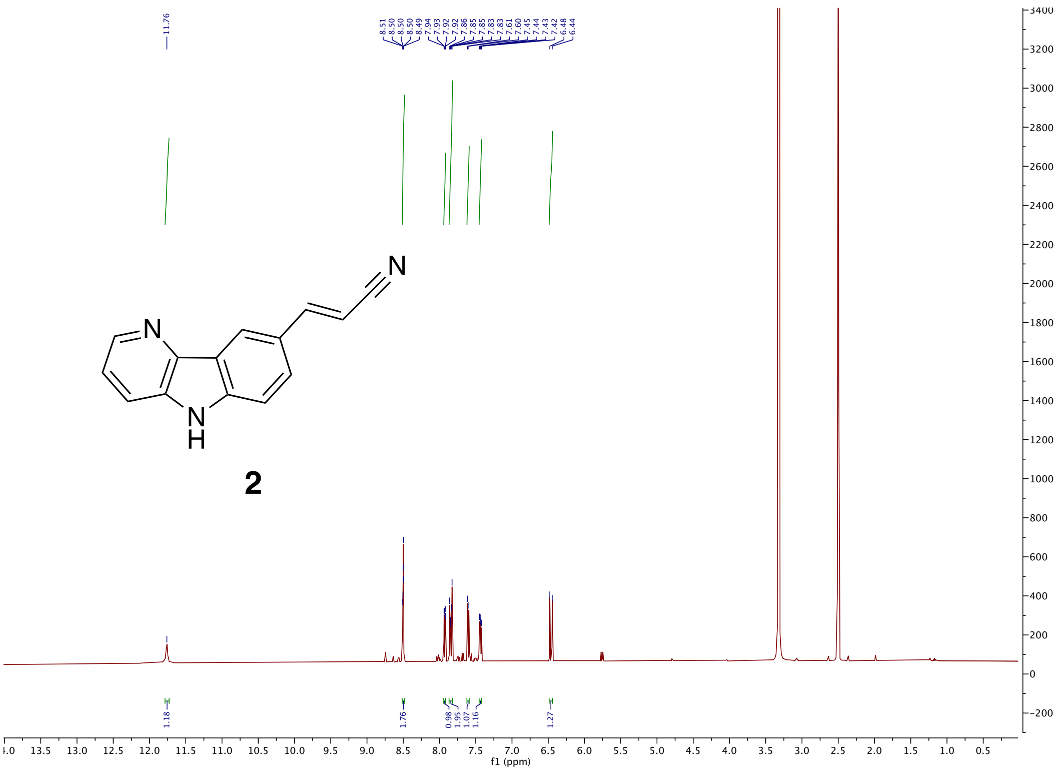

^{1}H NMR spectrum of **2** in DMSO-d_6 (500 Hz) at 25 °C.

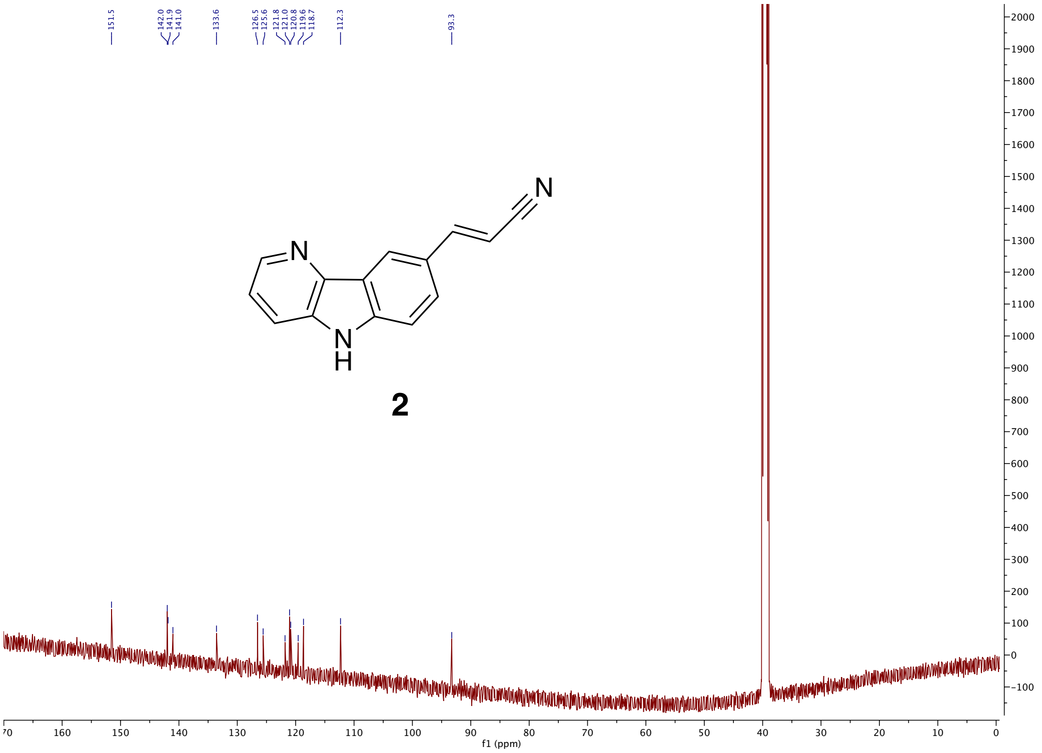

^{13}C NMR spectrum of **2** in DMSO-d_6 (125 Hz) at 25 °C.

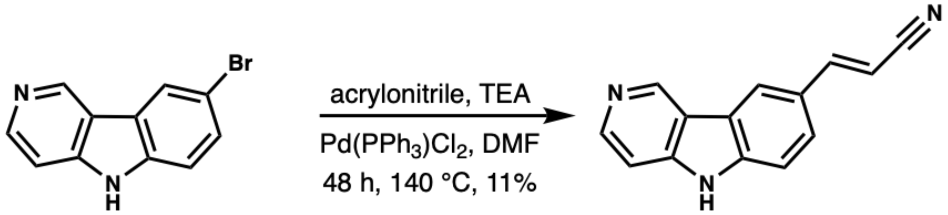

3

To a reaction flask was added 8-bromo-5*H*-pyrido[4,3-b]indole (50.8 mg, 0.21 mmol) and acrylonitrile (22.3 mg, 0.42 mmol) in DMF (2 mL). TEA (107.0 mg, 1.05 mmol) was then added to the reaction mixture, followed by the addition of bis(triphenylphosphine)palladium(II) dichloride (14.7 mg, 0.021 mmol). The reaction was stirred at room temperature for 20 minutes and was then heated to 140°C and stirred for 48 h. The mixture was then extracted with EtOAc (8 mL x 3) and washed with saturated $NaHCO_3$ and brine. The combined organic layers were dried over $MgSO_4$, filtered and concentrated *in vacuo*. The resulting residue was purified by ISCO flash column chromatography (0-100% ethyl acetate in hexanes) to yield **3** (5.0 mg, 11%) as a tan solid. ^{1}H NMR (500 MHz, DMSO-d_6): δ 13.33 (s, 1H), 9.72 (s, 1H), 8.72–8.70 (m, 2H), 8.05–8.04 (m, 1H), 8.02 (dd, J = 8.6, 1.5 Hz, 1H), 7.86 (d, J = 8.9 Hz, 1H), 7.84 (s, 1H), 6.59 (d, J = 16.6 Hz, 1H); ^{13}C NMR (125 MHz, DMSO-d_6): δ 150.6, 148.9, 147.3, 142.6, 136.0, 135.8, 128.5, 128.3,

122.4, 120.9, 119.7, 113.6, 108.9, 95.7; HRMS: (ESI+) m/z calculated for $C_{14}H_{10}N_3$ $[M+H]^+$: 220.0869, found: 220.0861.

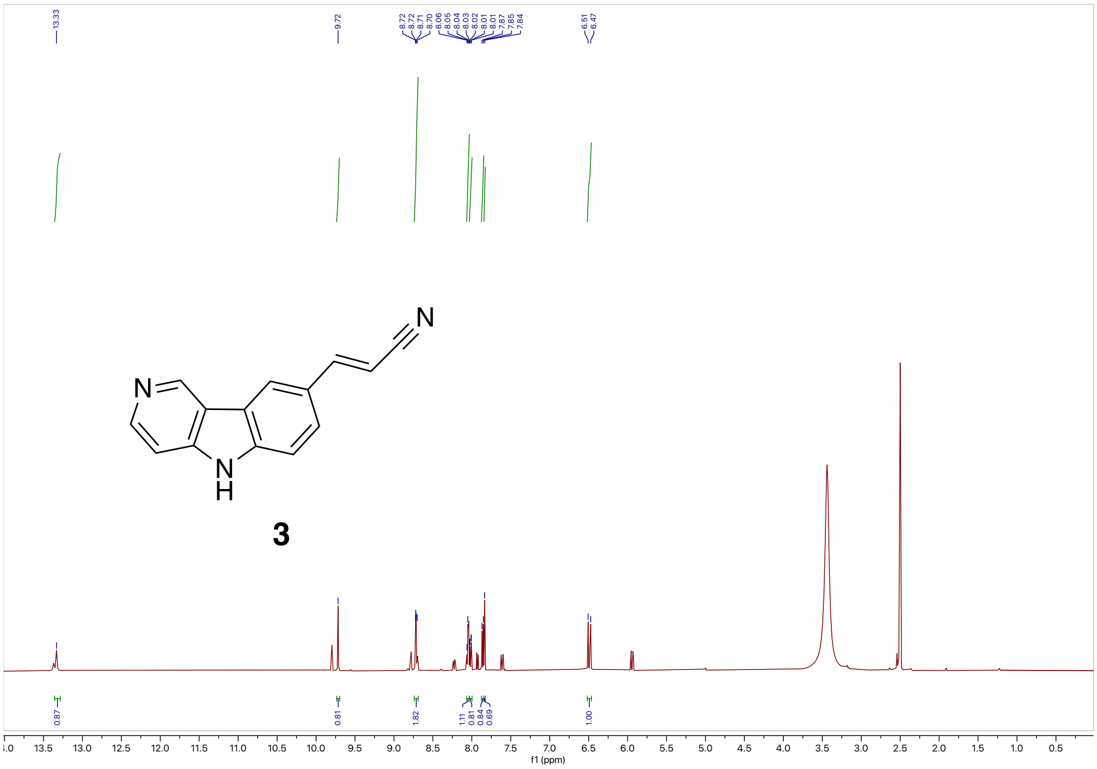

^1H NMR spectrum of **3** in DMSO-d_6 (500 Hz) at 25 °C.

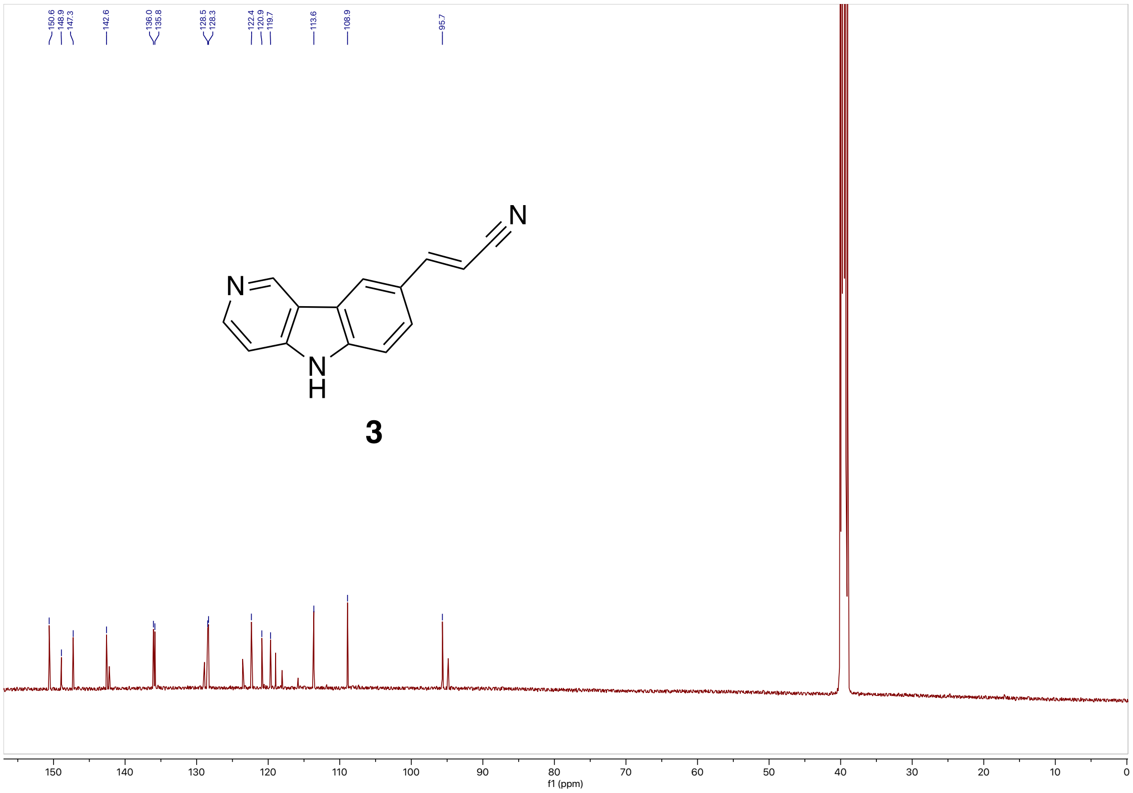

^{13}C NMR spectrum of **3** in DMSO-d_6 (125 Hz) at 25 °C.

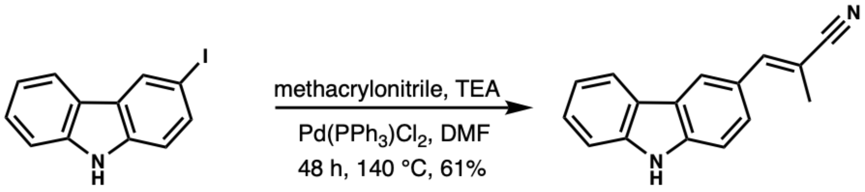

To a reaction flask was added 3-iodo-9*H*-carbazole (132.1 mg, 0.45 mmol) and methacrylonitrile (60.4 mg, 0.90 mmol) in DMF (3 mL). TEA (229.3 mg, 2.25 mmol) was then added to the reaction mixture, followed by the addition of bis(triphenylphosphine)palladium(II) dichloride (31.9 mg, 0.045 mmol). The reaction was stirred at room temperature for 20 minutes and was then heated to 140 °C and stirred for 48 h. The mixture was then extracted with EtOAc (8 mL x 3) and washed with saturated $NaHCO_3$ and brine. The combined organic layers were dried over $MgSO_4$, filtered and concentrated *in vacuo*. The resulting residue was purified by ISCO flash column chromatography (0-100% ethyl acetate in hexanes) to yield **4** (46.6 mg, 61%) as a white powder. ^{1}H NMR (500 MHz, DMSO-d_6): δ 11.56 (s, 1H), 8.44 (d, *J* = 1.7 Hz, 1H), 8.08 (d, *J* = 1.6 Hz, 1H), 7.87 (dd, *J* = 8.5, 1.7 Hz, 1H), 7.56 (d, *J* = 8.5 Hz, 1H), 7.52 (d, *J* = 8.1 Hz, 1H), 7.43 (m, 1H),

7.37 (d, J = 1.3 Hz, 1H), 7.21 (m, 1H), 2.15 (d, J = 1.3 Hz, 3H); ^{13}C NMR (125 MHz, DMSO-d_6): δ 145.3, 140.4, 140.3, 126.2, 125.5, 124.6, 122.5, 122.2, 121.1, 120.3, 120.2, 119.3, 111,4, 111.3, 101.4, 21.4; HRMS: (ESI+) m/z calculated for $C_{16}H_{13}N_2$ $[M+H]^+$: 233.1073, found: 233.1067.

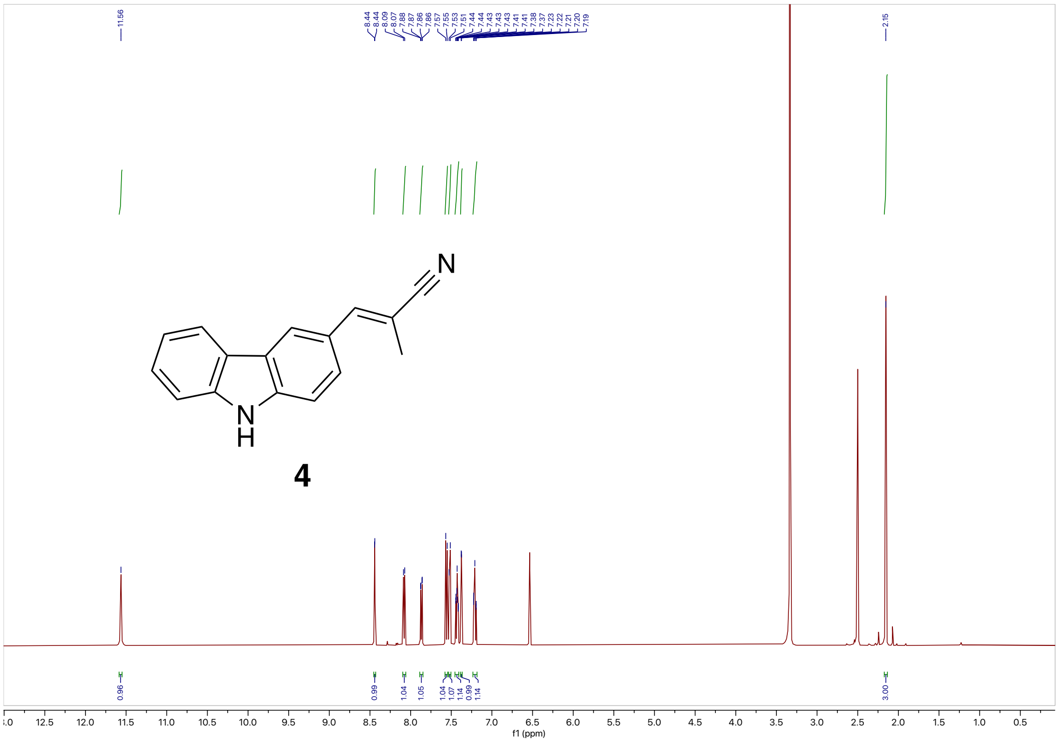

^1H NMR spectrum of **4** in DMSO-d_6 (500 Hz) at 25 °C.

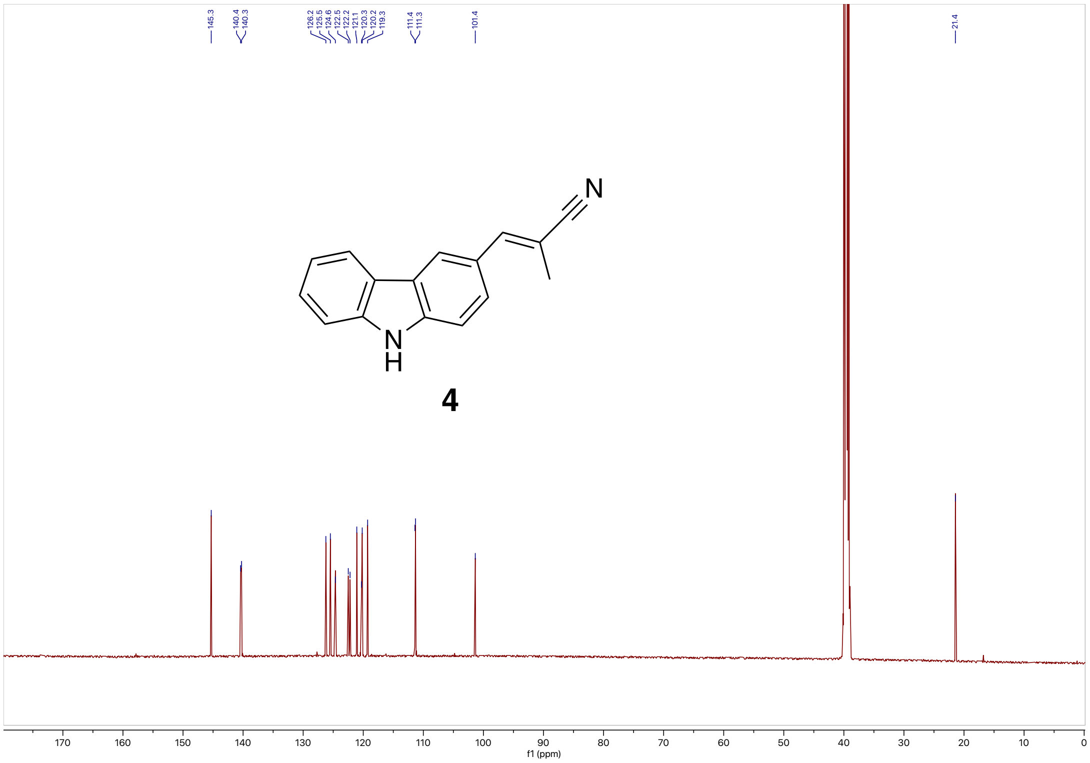

^{13}C NMR spectrum of **4** in DMSO-d_6 (125 Hz) at 25 °C.

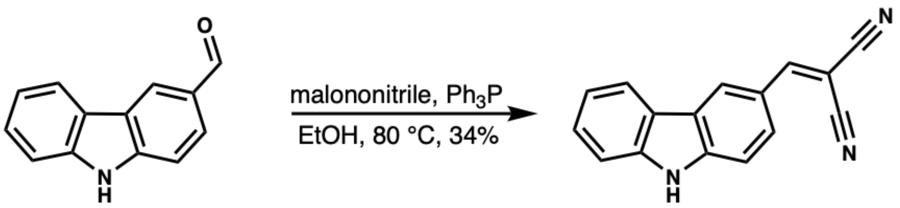

5

To a reaction flask was added 9*H*-carbazole-3-carbaldehyde (57.6 mg, 0.30 mmol), malononitrile (25.3 mg, 0.38 mmol) and triphenhylphosphine in EtOH (2 mL). The reaction was stirred at 80°C overnight. The solvent was evaporated *in vacuo*. The crude was then extracted with EtOAc (6 mL x 3) and washed with brine. The combined organic layers were dried over $MgSO_4$, filtered and concentrated *in vacuo*. The resulting residue was purified by ISCO flash column chromatography (0-100% ethyl acetate in hexanes) to yield **5** (24.6 mg, 34%) as an orange solid. ^{1}H NMR (500 MHz, DMSO-d_6): δ 12.10 (s, 1H), 8.75 (d, *J* = 1.6 Hz, 1H), 8.53 (s, 1H), 8.15–8.11 (m, 2H), 7.70 (d, *J* = 8.9 Hz, 1H), 7.60–7.58 (m,1H), 7.52–7.49 (m, 1H), 7.32–7.29 (m, 1H); ^{13}C NMR (125 MHz, DMSO-d_6): δ 161.7, 143.4, 140.5, 127.6, 127.2, 125.8, 122.9, 122.4, 122.1, 120.5, 120.5, 115.4, 114.7, 112.2, 112.0, 74.7; HRMS: (ESI+) m/z calculated for $C_{16}H_{10}N_3$ [M+H]$^+$: 243.0791, found: 243.0783.

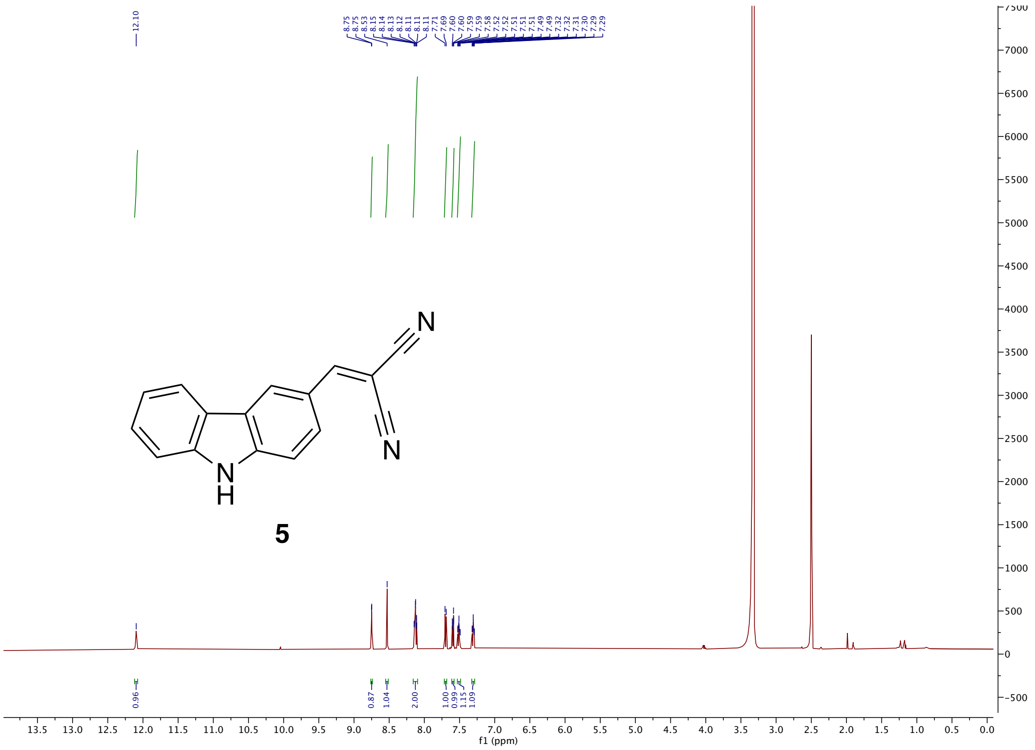

^{1}H NMR spectrum of **5** in DMSO-d_6 (500 Hz) at 25 °C.

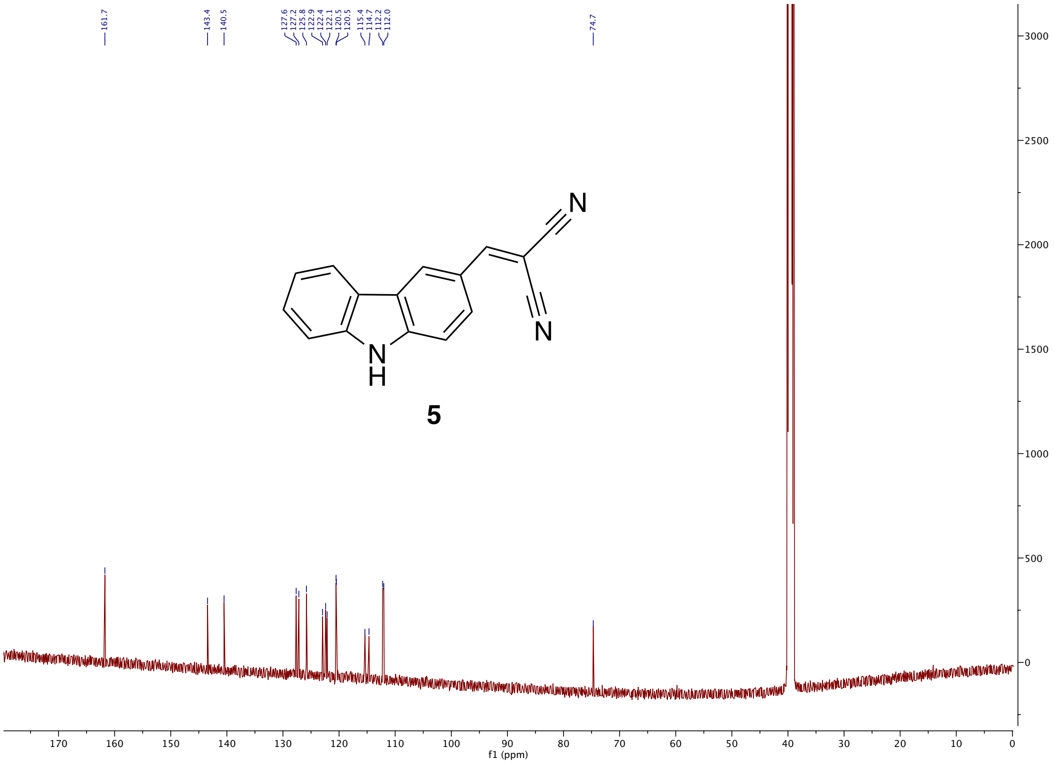

^{13}C NMR spectrum of **5** in DMSO-d_6 (125 Hz) at 25 °C.

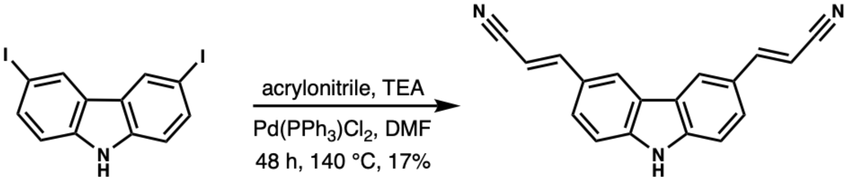

To a reaction flask was added 3,6-diiodo-9*H*-carbazole (259.6 mg, 0.62 mmol) and acrylonitrile (131.5 mg, 2.48 mmol) in DMF (4 mL). TEA (366.9 mg, 3.6 mmol) was then added to the reaction mixture, followed by the addition of bis(triphenylphosphine)palladium(II) dichloride (43.5 mg, 0.06 mmol). The reaction was stirred at room temperature for 20 minutes and was then heated to 140 °C and stirred for 48 h. The mixture was then extracted with EtOAc (8 mL x 3) and washed with saturated $NaHCO_3$ and brine. The combined organic layers were dried over $MgSO_4$, filtered and concentrated *in vacuo*. The resulting residue was purified by ISCO flash column chromatography (0-100% ethyl acetate in hexanes) to yield **6** (28.90 mg, 17%) as a tan solid. ^{1}H NMR (500 MHz, DMSO-d_6): δ 11.93 (s, 1H), 8.41 (d, *J* = 1.6 Hz, 2H), 7.79 (d, *J* = 6.7 Hz, 2H), 7.77 (dd, *J* = 8.5, 1.6 Hz, 2H), 7.56 (d, *J* = 8.5 Hz, 1H), 6.38 (d, *J* = 6.7 Hz, 1H); ^{13}C NMR (125 MHz, DMSO-d_6): δ 151.6, 141.9, 125.7, 125.6, 122.6, 121.2, 119.5, 112.0, 93.1; HRMS: (ESI+) m/z calculated for $C_{18}H_{12}N_3$ [M+H]$^+$: 270.1026, found: 270.1021.

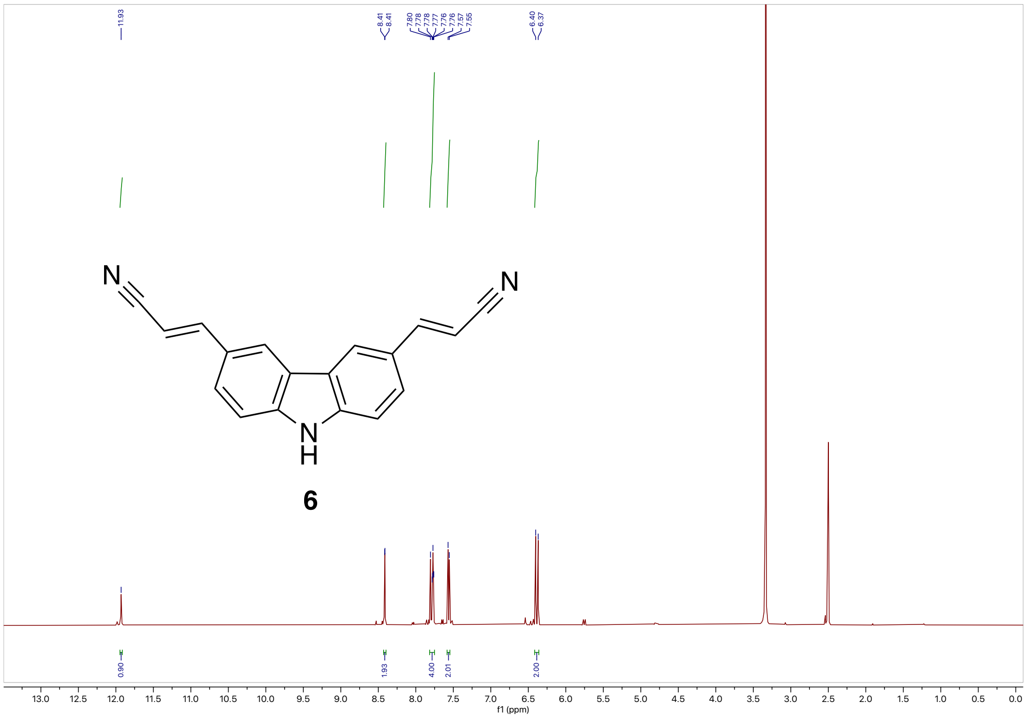

^{1}H NMR spectrum of **6** in DMSO-d_6 (500 Hz) at 25 °C.

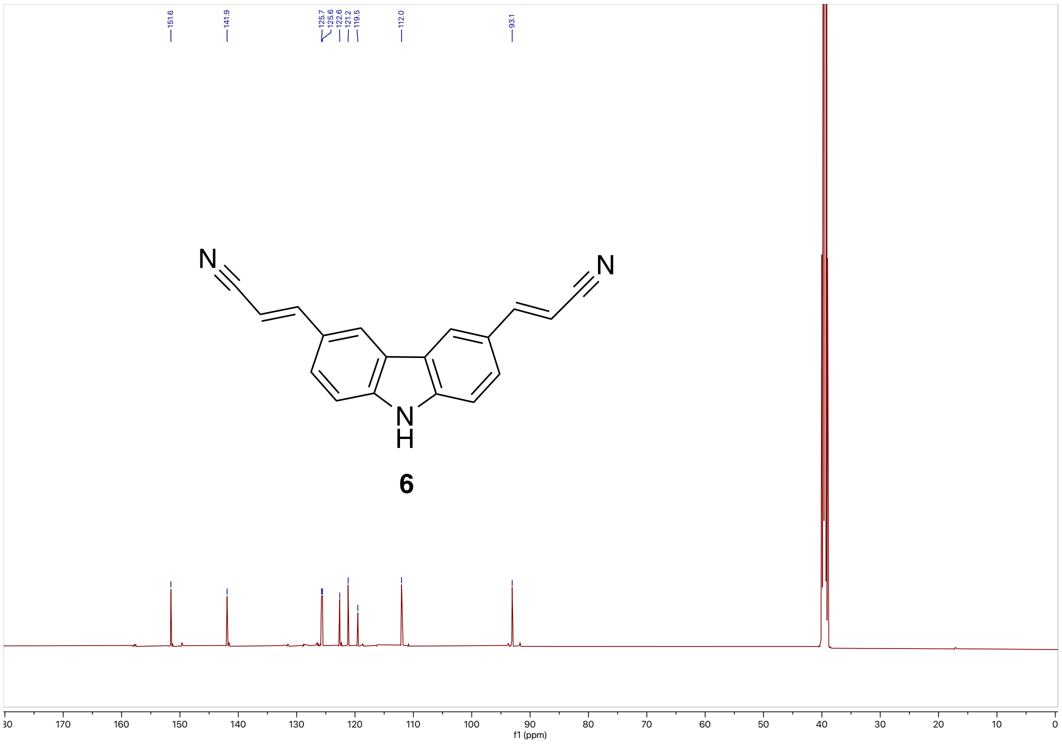

^{13}C NMR spectrum of **6** in DMSO-d_6 (125 Hz) at 25 °C.

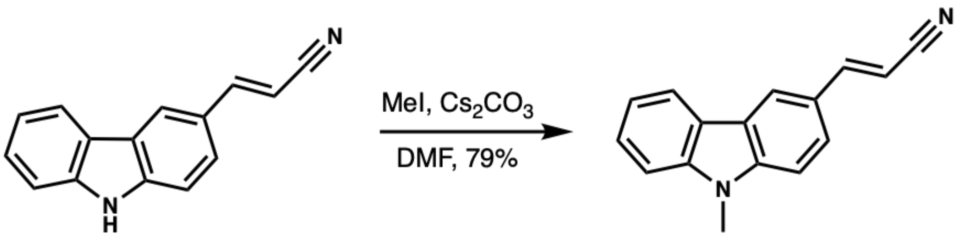

1 **7**

To a reaction flask was added **1** (20.0 mg, 0.09 mmol) and Cs_2CO_3 (44.8 mg, 0.14 mmol) in DMF (0.5 mL). MeI (19.5 mg, 0.14 mmol) was then added to the solution. The reaction was stirred at room temperature overnight. The solvent was removed *in vacuo* and the resulting residue was purified by ISCO flash column chromatography (0-100% ethyl acetate in hexanes) to yield **7** (16.91 mg, 79%) as a white solid. ^{1}H NMR (500 MHz, DMSO-d_6): δ 8.49 (s, 1H), 8.15 (d, *J* = 7.7 Hz, 1H), 7.79 (m, 2H), 7.65 (m, 2H), 7.52 (m, 1H), 7.27 (t, *J* = 7.5 Hz, 1H), 6.41 (d, *J* = 16.7 Hz, 1H), 3.91 (s, 3H); ^{13}C NMR (125 MHz, DMSO-d_6): δ 151.7, 142.1, 141.2, 126.5, 125.5, 124.9, 122.3, 121.9, 120.8, 120.4, 119.7, 109.7, 109.7, 92.5, 48.6, 29.2; HRMS: (ESI+) m/z calculated for $C_{16}H_{13}N_2$ $[M+H]^+$: 233.2935, found: 233.2940.

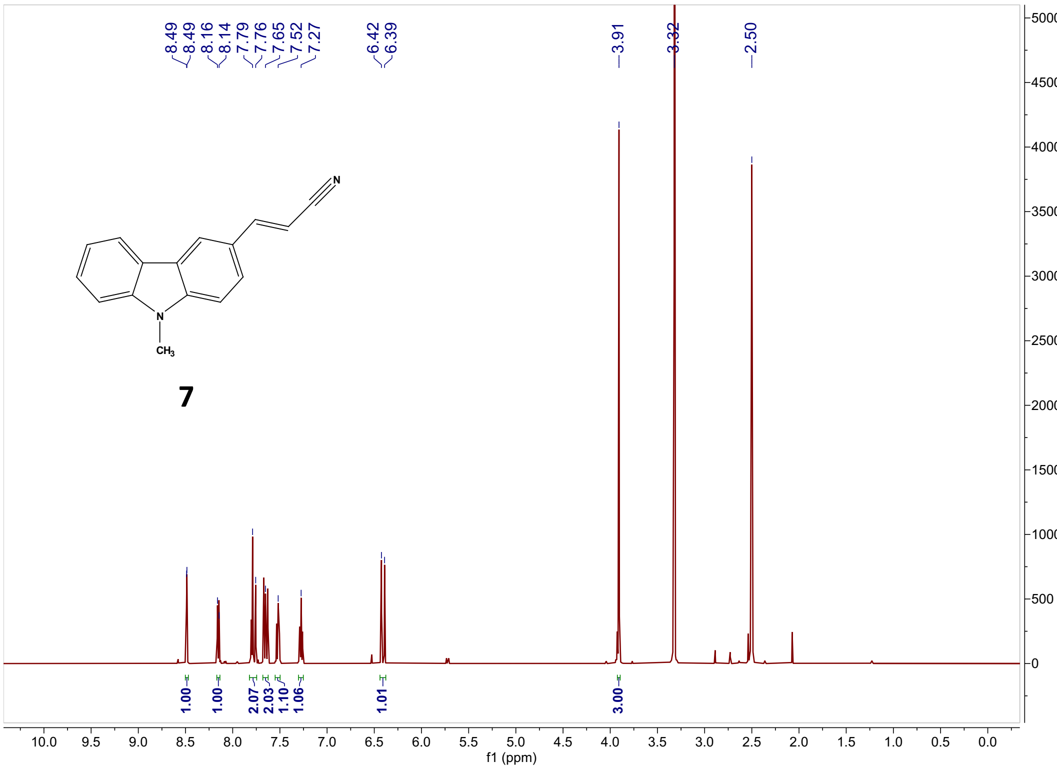

^{1}H NMR spectrum of **7** in DMSO-d_6 (500 Hz) at 25 °C.

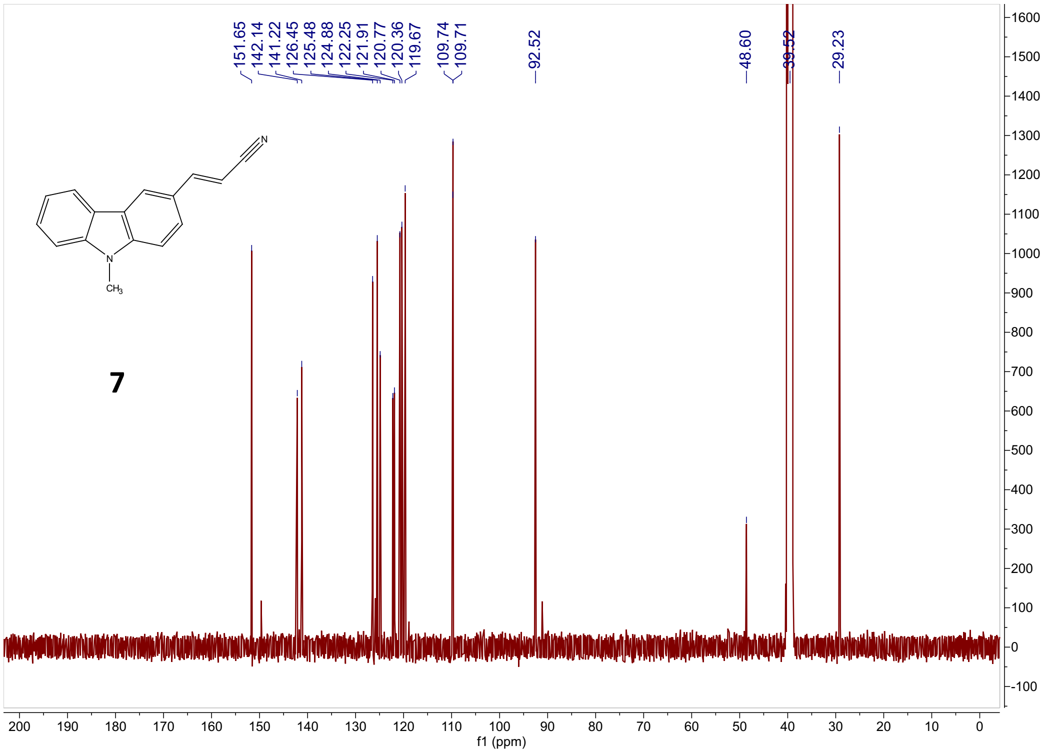

^{13}C NMR spectrum of **7** in DMSO-d_6 (500 Hz) at 25 °C.

Synthesis of biotinylated probe

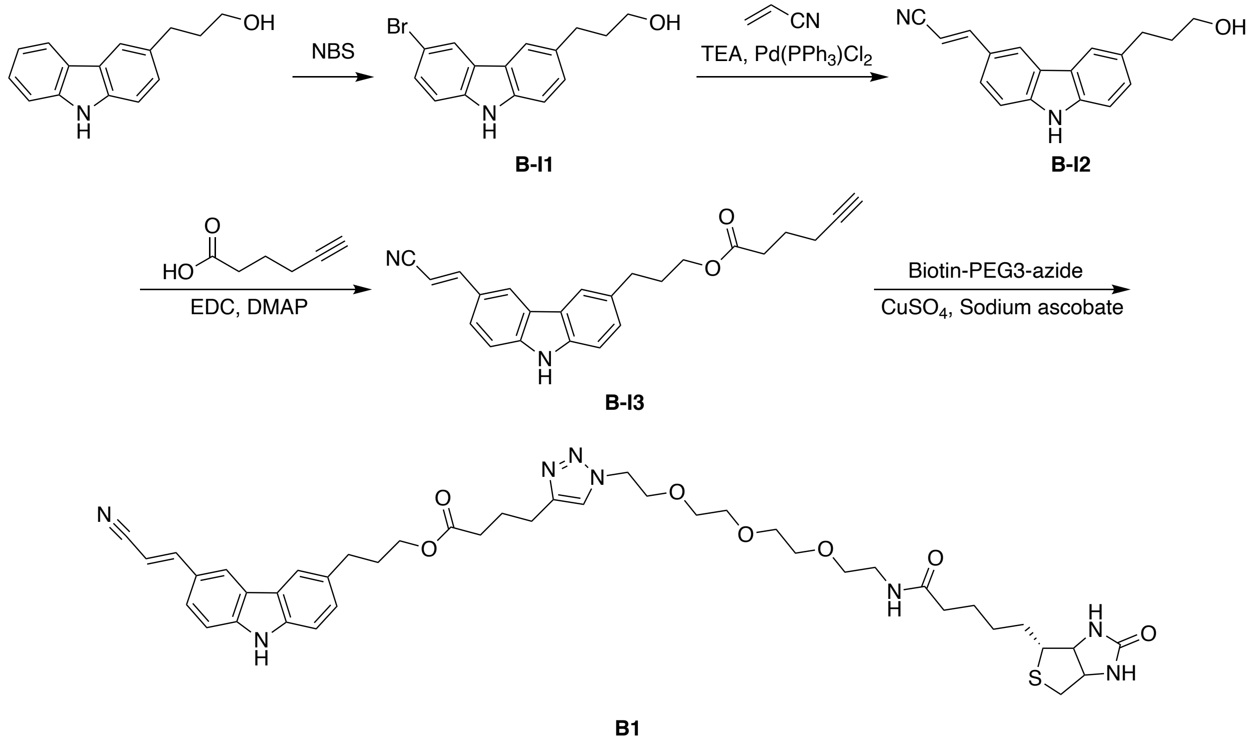

To a reaction flask was added 3-(9*H*-carbazol-3-yl)propan-1-ol (208.2 mg, 0.92 mmol) in anhydrous acetonitrile (8 mL). A solution of NBS (164.4 mg, 0.92 mmol) in DMF (0.5 mL) was added to the reaction flask dropwise. The reaction was stirred at room temperature for 2 h and was purified by ISCO flash column chromatography (0-100% ethyl acetate in hexanes) to yield **B-I1** (245.5 mg, 88%) as a tan solid. ^{1}H NMR (500 MHz, DMSO-d_6): δ 11.28 (s, 1H), 8.30 (d, *J* = 1.6 Hz, 1H), 7.96 (s, 1H), 7.46 (dd, *J* = 8.6, 1.9 Hz, 2H), 7.40 (m, 2H), 7.26 (dd, *J* = 8.3, 1.4 Hz, 2H), 4.47 (t, *J* = 4.6 Hz, 1H), 3.45 (dt, *J* = 6.1, 5.4 Hz, 2H), 2.75 (t, *J* = 7.7 Hz, 1H), 1.80 (quint, *J* = 6.5 Hz, 2H); ^{13}C NMR (125 MHz, DMSO-d_6): δ 139.1, 139.0, 133.2, 128.1, 127.6, 124.8, 123.1, 122.0, 120.3, 113.3, 111.4, 110.7, 60.6, 35.5, 32.2; HRMS: (ESI+) m/z calculated for $C_{18}H_{12}N_3$ $[M+H]^+$: 270.1026, found: 270.1021.

To a reaction flask was added **B-I1** (231.8 mg, 0.76 mmol), acrylonitrile (80.6 mg, 1.52 mmol) in DMF (3 mL). TEA (387.7 mg, 3.80 mmol) was then added to the reaction mixture, followed by the addition of bis(triphenylphosphine)palladium(II) dichloride (53.3 mg, 0.076 mmol). The reaction was stirred at room temperature for 20 minutes and was then heated to 140 °C and stirred for 48 h. The reaction residue was extracted with EtOAc (20 mL × 3) and washed with saturated $NaHCO_3$ and brine. The combined organic layers were dried over $MgSO_4$, filtered and concentrated *in vacuo*. The resulting residue was purified by ISCO flash column chromatography (0-100% ethyl acetate in hexanes) to yield **B-I2** (58.3 mg, 28%) as an off-white solid. ^{1}H NMR (500 MHz, DMSO-d_6): δ 11.54* (s, 1H), 11.47 (s, 1H), 8.53* (d, *J* = 1.3 Hz, 1H), 8.42 (d, *J* = 1.2 Hz, 1H), 8.10* (d, *J* = 8.0 Hz, 1H), 7.98* (dd, *J* = 8.6, 1.7 Hz, 1H), 7.91 (d, *J* = 14.8 Hz, 1H), 7.74* (d, *J* = 17.6 Hz, 1H), 7.67 (dd, *J* = 8.5, 1.7 Hz, 1H), 7.56 (d, *J* = 8.5 Hz, 1H), 7.49–7.41 (m, 2H), 7.49–7.41* (m, 2H), 7.30–7.26* (m, 1H), 7.30–7.26 (m, 1H), 6.35 (d, *J* = 16.6 Hz, 1H), 5.68* (d, *J* = 12.1 Hz, 1H), 4.49–4.47 (m, 1H), 3.47–3.44 (m, 2H), 2.79–2.76 (m, 2H), 1.83–1.78 (m, 2H); ^{13}C NMR (125 MHz, DMSO-d_6): δ 151.9, 149.9*, 141.7, 141.3*, 138.7*, 138.7, 133.3*, 133.2, 125.7*, 125.2, 124.6*, 124.5, 122.6, 122.4*, 122.4*, 122.3, 122.2*, 120.8, 119.5, 119.4*, 119.0, 111.3*, 111.3, 111.3, 111.2*, 92.0, 90.5*, 60.1, 35.1*, 35.1, 31.7; **Z* isomer, *E*/*Z* ratio = 1:1; HRMS: (ESI+) m/z calculated for $C_{15}H_{11}N_2$ $[M+H]^+$: 219.0917, found: 219.0909.

To a reaction flask was added **B-I2** (27.7 mg, 0.100 mmol), 5-hexynoic acid (12.4 mg, 0.110 mmol) in THF (1 mL) and put under N_2 atmosphere. Next, EDC (57.6 mg, 0.301 mmol) was added to the reaction mixture followed by addition of catalytic DMAP (1.22 mg, 0.010 mmol). The resulting reaction mixture was allowed to stir overnight at room temperature. Afterwards, the reaction residue was then concentrated *in vacuo* and the resulting residue was purified by ISCO

flash column chromatography (0-60% ethyl acetate in hexanes) to yield **B-I3** (26.3 mg, 71%) as an off-white solid. ^{1}H NMR (500 MHz, DMSO-d_6): δ 11.56* (s, 1H), 11.50 (s, 1H), 8.52* (d, *J* = 1.4 Hz, 1H), 8.42 (d, *J* = 1.4 Hz, 1H), 7.98* (dd, *J* = 8.6, 1.9 Hz, 1H), 7.93 (d, *J* = 12.8 Hz, 1H), 7.74* (d, *J* = 16.6 Hz, 1H), 7.68 (dd, *J* = 8.5, 1.7 Hz, 1H), 7.57 (d, *J* = 8.6 Hz, 1H), 7.49–7.43 (m, 2H), 7.49–7.43* (m, 2H), 7.31–7.27* (m, 1H), 7.31–7.27 (m, 1H), 6.35 (d, *J* = 16.6 Hz, 1H), 5.68* (d, *J* = 12.1 Hz, 1H), 4.07–4.04 (m, 1H), 2.83–2.79 (m, 3H), 2.42 (t, *J* = 7.3 Hz, 2H), 2.22–2.18 (m, 2H), 2.00–1.94 (m, 2H), 1.73–1.67 (m, 2H); ^{13}C NMR (125 MHz, DMSO-d_6): δ 172.5, 151.9, 149.9*, 141.7, 141.3*, 138.9*, 138.8, 132.3*, 132.2, 127.1*, 127.0, 125.7*, 125.2, 124.6*, 124.5, 122.6, 122.5*, 122.4*, 122.4, 122.2*, 120.8, 119.6, 119.5*, 118.9, 111.4, 111.3*, 92.1, 90.6*, 83.7, 71.8, 71.8*, 63.3, 32.3, 31.5*, 31.5, 30.5, 30.5*, 23.5, 17.1; **Z* isomer, *E*/*Z* ratio = 1:1; HRMS: (ESI+) m/z calculated for $C_{24}H_{22}N_2O_2$ $[M+H]^+$: 371.1755, found: 371.1750.

To a reaction flask under N_2 gas was added **B-I3** (26.3 mg, 0.070 mmol), Biotin-PEG3-Azide (34.7 mg, 0.078 mmol) in DMF (0.5 mL). Next, a 0.2 M aqueous solution of CuSO4 (2.27 mg, 0.014 mmol) was added to the reaction mixture followed by a 0.2M aqueous solution of Na ascorbate (2.81 mg, 0.014 mmol). The resulting reaction mixture was allowed to stir overnight at room temperature under N_2 atmosphere. Afterwards, the reaction was then concentrated *in vacuo* and the resulting residue was purified by ISCO ACCQPrep reverse phase column chromatography (10-95% acetonitrile in water) to yield **B1** (5213 mg, 37%) as an off-white solid. ^{1}H NMR (500 MHz, DMSO-d_6): δ 11.56* (s, 1H), 11.49 (s, 1H), 8.51* (s, 1H), 8.42 (s, 1H), 8.10* (d, *J* = 8.0 Hz, 1H), 7.99* (d, *J* = 8.7 Hz, 1H), 7.93 (d, *J* = 12.5 Hz, 1H), 7.83–7.80 (m, 2H), 7.74* (d, *J* = 17.6 Hz, 1H), 7.68 (d, *J* = 8.6, Hz, 1H), 7.56 (d, *J* = 8.6 Hz, 1H), 7.49–7.42 (m, 2H), 7.30–7.27 (m, 1H), 6.40 (s, 1H), 6.34 (s, 1H), 6.34 (d, *J* = 16.4 Hz, 1H), 5.68* (d, *J* = 12.1 Hz, 1H), 4.47 (t, *J* = 5.4 Hz, 2H), 4.30–4.27 (m, 1H), 4.12–4.09 (m, 1H), 4.05 (t, *J* = 6.4 Hz, 2H), 3.78 (t, *J* = 5.1 Hz, 2H), 3.51–3.49 (m, 2H), 3.18–3.15 (m, 3H), 2.81–2.78 (m, 3H), 2.65–2.62 (m, 2H), 2.40–2.36 (m, 2H), 2.04 (t, *J* = 7.2 Hz, 2H), 2.00–1.94 (m, 2H), 1.88–1.84 (m, 2H), 1.60–1.57 (m, 1H), 1.49–1.42 (m, 3H), 1.30–1.23 (m, 3H); ^{13}C NMR (125 MHz, DMSO-d_6): δ 195.8, 172.8, 167.8, 162.7, 146.0, 137.6, 126.1, 125.9, 124.6, 122.5, 122.5, 122.4, 122.4, 121.5, 121.3, 121.3, 121.2, 121.1, 120.9, 119.7, 119.7, 111.4, 69.7, 69.6, 69.6, 69.2, 68.8, 63.3, 61.1, 59.2, 55.4, 49.2, 48.6, 38.4, 35.1, 32.9, 28.2, 28.0, 25.3, 24.4, 24.3; HRMS: (ESI+) m/z calculated for $C_{42}H_{54}N_8O_7S$ $[M+H]^+$: 815.3909, found: 815.3915.

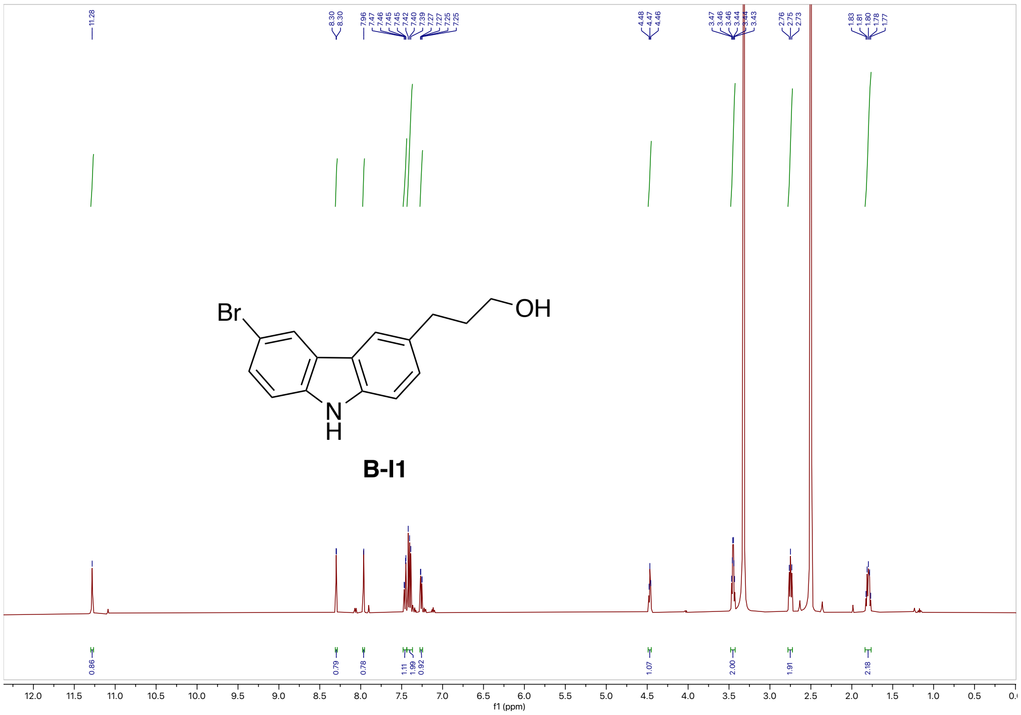

^{1}H NMR spectrum of **B-I1** in DMSO-d_6 (500 Hz) at 25 °C.

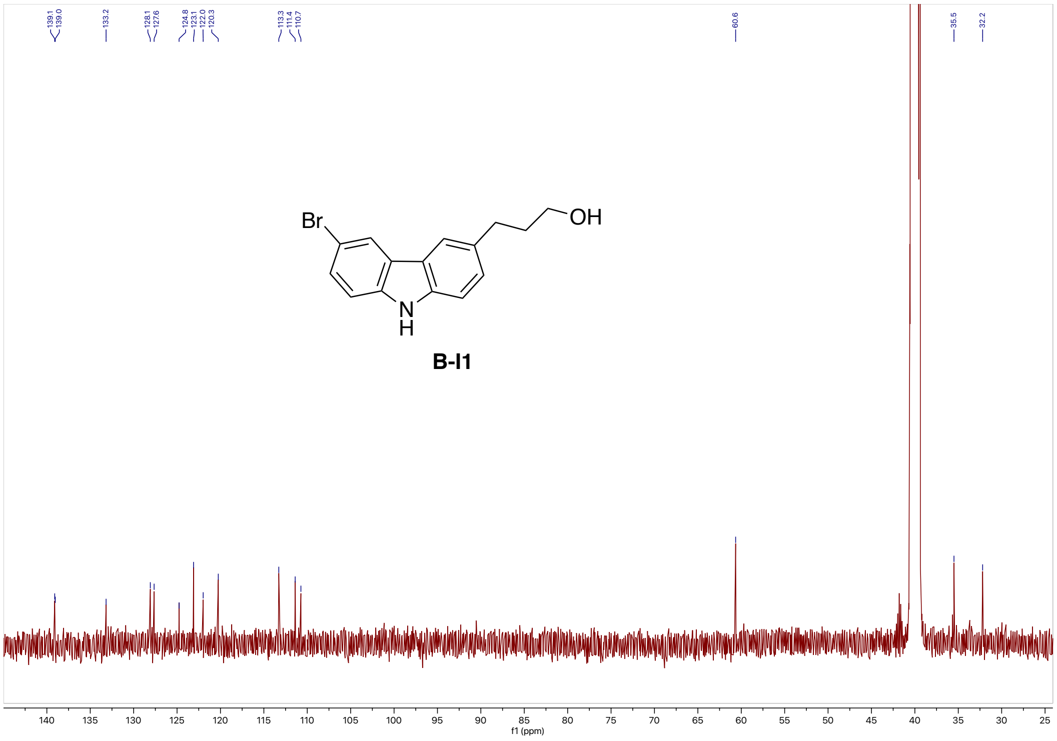

^{13}C NMR spectrum of **B-I1** in DMSO-d_6 (125 Hz) at 25 °C

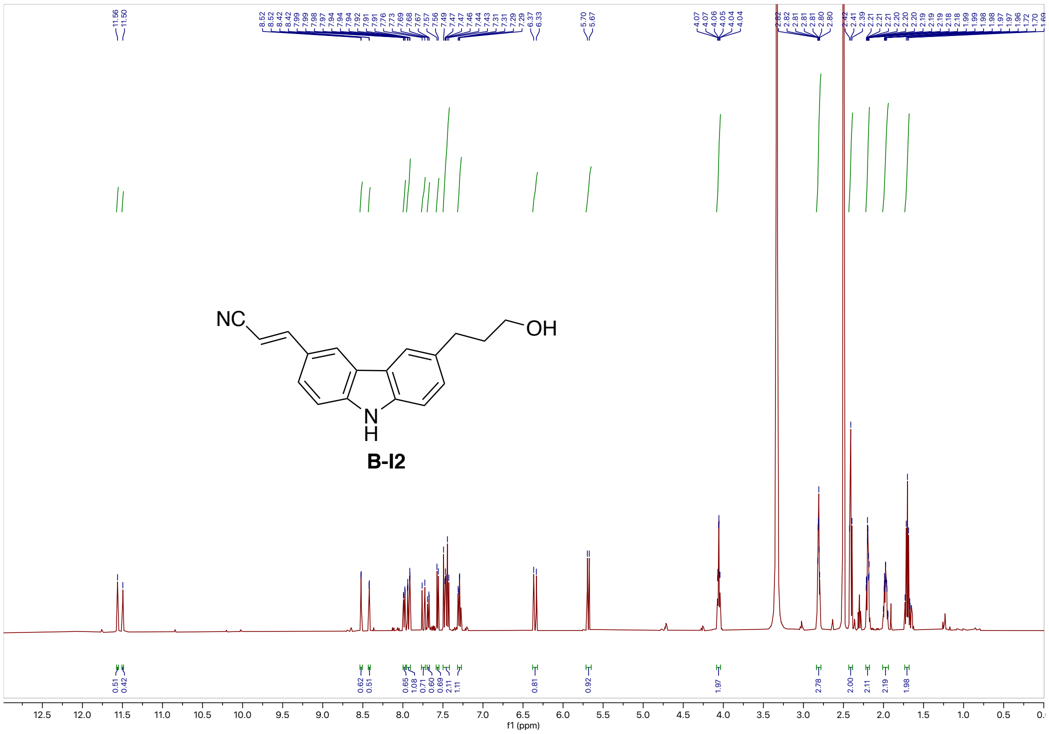

(a) ^{1}H NMR spectrum of **B-I2** in DMSO-d_6 (500 Hz) at 25 °C.

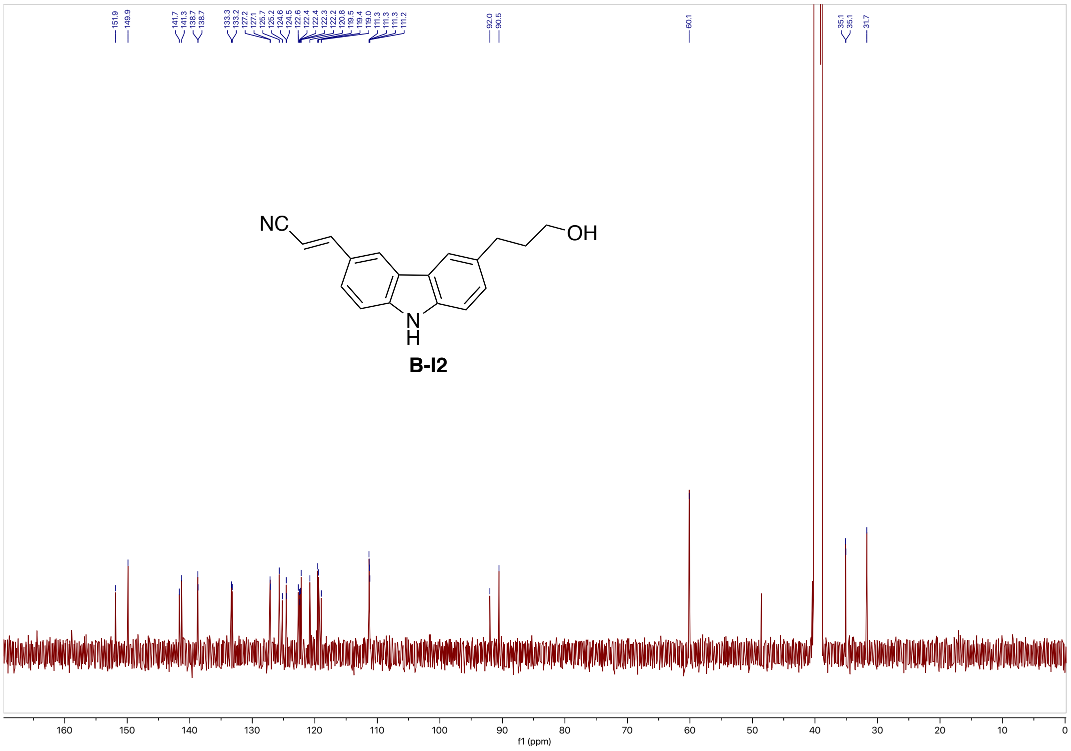

^{13}C NMR spectrum of **B-I2** in DMSO-d_6 (125 Hz) at 25 °C.

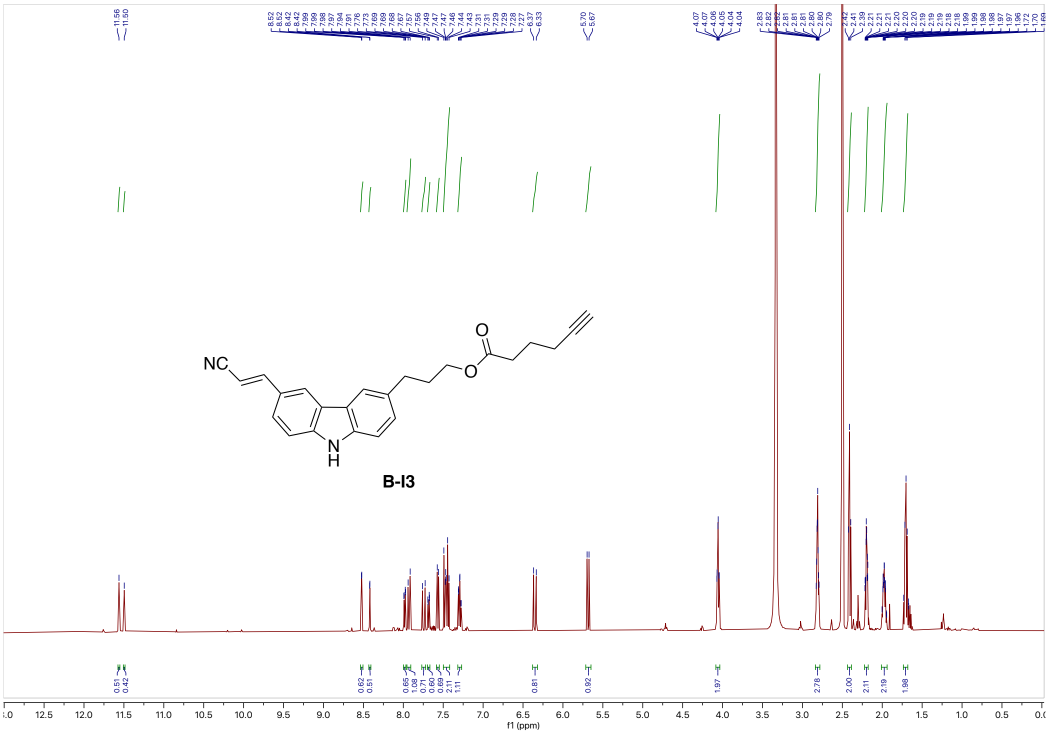

^{1}H NMR spectrum of **B-I3** in DMSO-d_6 (500 Hz) at 25 °C.

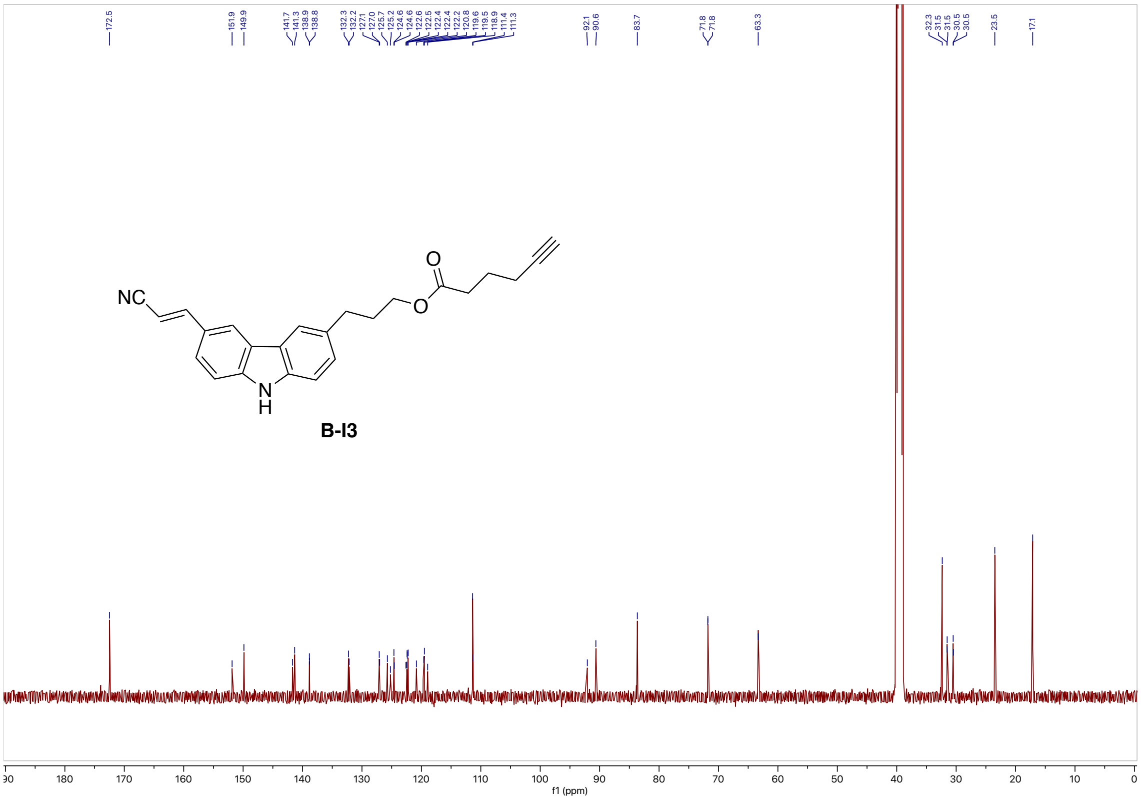

^{13}C NMR spectrum of **B-I3** in DMSO-d_6 (125 Hz) at 25 °C

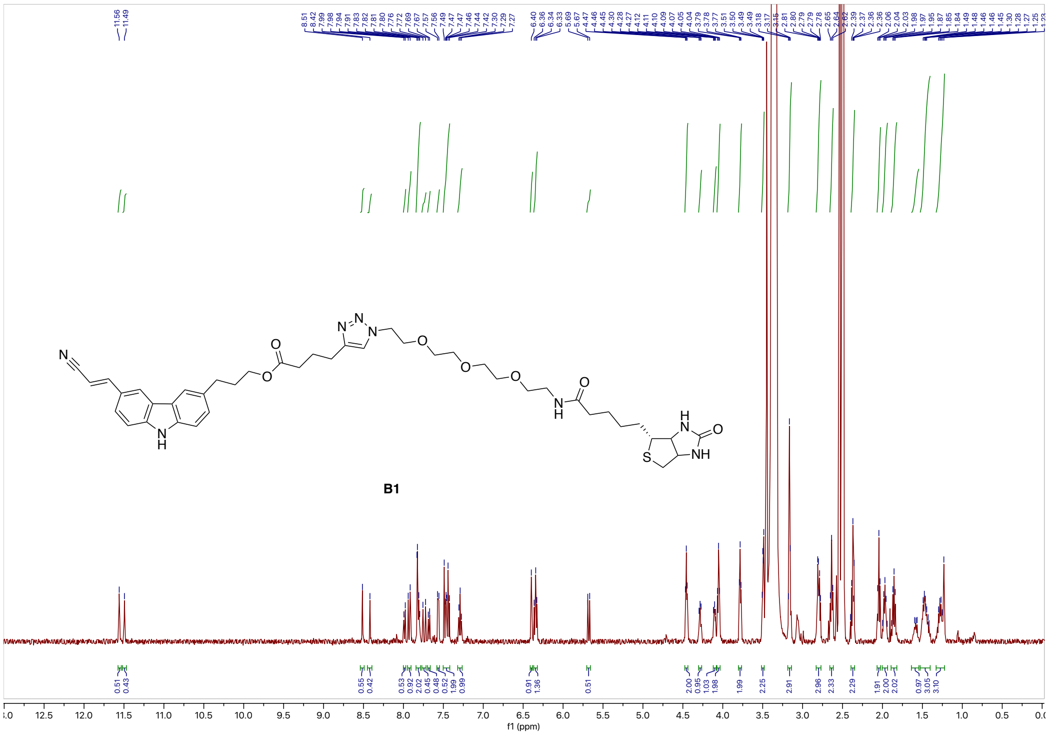

^{1}H NMR spectrum of **B1** in DMSO-d_6 (500 Hz) at 25 °C.

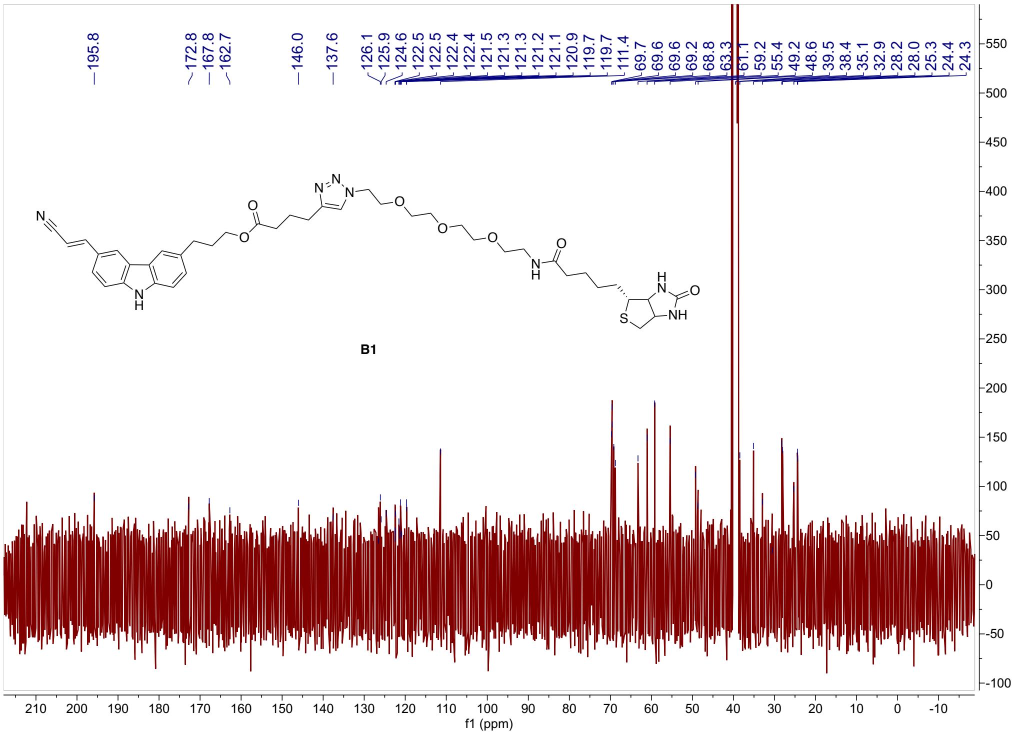

^{13}C NMR spectrum of **B1** in DMSO-d_6 (125 Hz) at 25 °C.
